# Fluid shear stress as a co-stimulatory cue to enhance T cell priming and restore activation in cancer patient T cells

**DOI:** 10.1093/jimmun/vkag166

**Published:** 2026-08-06

**Authors:** Nicole S Sarna, Monika Antunovic, Paula J Hurley, Kerry R Schaffer, Michael R King

**Affiliations:** Department of Bioengineering, Rice University, Houston, TX, United States; Department of Biomedical Engineering, Vanderbilt University, Nashville, TN, United States; Department of Medicine, Vanderbilt University Medical Center, Nashville, TN, United States; Vanderbilt-Ingram Cancer Center, Nashville, TN, United States; Department of Medicine, Vanderbilt University Medical Center, Nashville, TN, United States; Vanderbilt-Ingram Cancer Center, Nashville, TN, United States; Department of Medicine, Vanderbilt University Medical Center, Nashville, TN, United States; Vanderbilt-Ingram Cancer Center, Nashville, TN, United States; Geriatric Research Educational and Clinical Center (GRECC), VA Tennessee Valley Healthcare System, Nashville, TN, United States; Department of Bioengineering, Rice University, Houston, TX, United States; Department of Biomedical Engineering, Vanderbilt University, Nashville, TN, United States

**Keywords:** immunotherapy, mechanobiology, prostate cancer, T cell activation

## Abstract

Cancer patient-derived T cells often exhibit impaired activation and functional responsiveness due to chronic antigen exposure and therapy-induced immune dysregulation, limiting the efficacy of current immunotherapies. Mechanical forces, such as fluid shear stress (FSS), are emerging as critical regulators of immune cell activation, yet their role in modulating activation of patient-derived T cells remains largely unexplored. In this study, primary T cells isolated from patients with metastatic prostate cancer were exposed to FSS using a cone-and-plate viscometer during ex vivo activation in the presence and absence of bead-bound anti-CD3/CD28 monoclonal antibodies. FSS alone was sufficient to induce NF-κB phosphorylation and intracellular cytokine synthesis, demonstrating that mechanical stimulation can independently initiate T cell activation signaling. Moreover, FSS combined with anti-CD3/CD28 stimulation produced a synergistic increase in activation signaling, IL-2 and IFN-γ production, and CD25/CD69 expression. While trends were consistent across donors overall, inter-patient variability reflected differences in baseline T cell phenotype, with naïve-like cells displaying greater mechanosensitivity. These results indicate that cancer patient-derived T cells retain mechanotransduction capacity despite reduced antigen responsiveness. Incorporating FSS as a co-stimulatory signal during ex vivo expansion may be more effective at priming patient T cells for activation, enhancing effector function, and improving the efficacy of adoptive cell therapies.

## Graphical Abstract

**Graphical Abstract:**
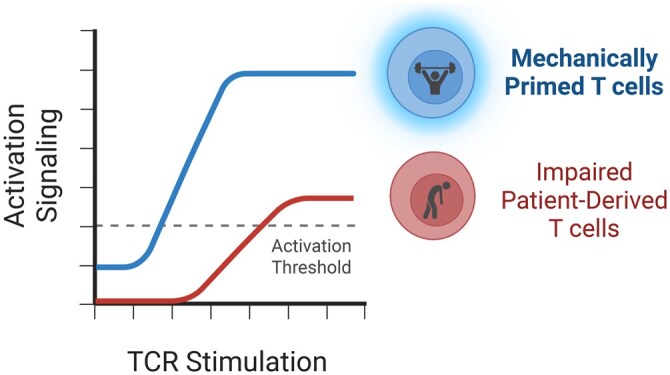

## Introduction

Prostate cancer remains the most commonly diagnosed cancer in men and represents the second leading cause of cancer-related deaths.^1^ In the United States, localized prostate cancer has a 5-year survival rate that exceeds 99% with surgery and/or radiation. Despite the excellent prognosis for localized disease, the 5-year survival rate falls to approximately 31% once metastatic.^2^ Current treatment options for advanced prostate cancer include hormone therapies, chemotherapies, radiopharmaceuticals, therapies that target DNA repair pathways, and immunotherapies.^3^^,^^4^ While androgen deprivation therapy (ADT) is largely successful initially alone and in combination with other classes of therapies for advanced prostate cancer management, nearly all prostate cancers become resistant to these therapies within the first few years of treatment. Despite recent advances in therapies for metastatic castration-resistant prostate cancer (mCRPC), this remains a terminal condition with poor prognosis, highlighting the need for continued research and development of novel therapeutics.

Prostate tumors are traditionally regarded as immunologically “cold,” with low T cell infiltration and an immunosuppressive tumor microenvironment (TME) that prevents effective antitumor responses.^5^ To date, checkpoint inhibitors have demonstrated minimal success in prostate cancer patients; only the 3%–6% of patients with high tumor mutational burden, mismatch repair deficiency or microsatellite instability (MSI)-high status appear to benefit.^6–8^ The immense success of immunotherapies and potential for durable benefit seen in other cancer types underscores the importance of continued efforts to study methods to engage and prime the immune system in prostate cancer.

Immune activation is orchestrated through a variety biochemical signaling cascades initiated by receptor-ligand interactions and co-stimulation, such as T cell receptor (TCR) engagement and CD28 signaling, that coordinate cellular communication and inflammatory regulation. However, emerging research has also highlighted the importance of mechanotransduction as a potent regulator of immune cell behavior.^9–11^ Our lab has shown that exposing primary immune cells from healthy donors, namely T cells,^12^^,^^13^ dendritic cells (DCs),^14^ and neutrophils,^15^ to fluid shear stress (FSS) significantly enhances and sustains activation signaling and effector function. We have further demonstrated this effect of FSS on mCRPC patient-derived DCs,^16^ but its potential application to patient-derived T cells remains unknown. In the context of mCRPC, patients typically undergo extensive therapeutic regimens and T cells are continually exposed to tumor antigens, where these factors are likely to impact T cell mechanosensitivity and activation thresholds.^17^^,^^18^ Investigating how FSS influences T cell activation in patient-derived cells is crucial for understanding the mechanobiology of T cells within the tumor microenvironment and could inform the development of more effective immunotherapies for this advanced form of disease.

In this study, we aimed to characterize the functional response of mCRPC patient-derived T cells to FSS during ex vivo activation using a cone-and-plate viscometer to create a uniform shearing flow. Our findings reveal that FSS alone is sufficient to initiate early T cell signaling and partially activate effector functions, suggesting a valuable role for mechanical stimuli in T cell priming. Additionally, we observed patient-specific differences in T cell phenotypes and memory acquisition under FSS conditions, emphasizing how inter-patient variability affects mechanosensitivity and activation potential. These findings demonstrate that patient-derived T cells remain mechanosensitive despite impaired antigen responsiveness and support the incorporation of biomechanical cues into ex vivo T cell manufacturing strategies to improve the efficacy and potential of T-cell based immunotherapies.

## Materials and methods

### Reagents and antibodies

RPMI-1640 with L-glutamine, fetal bovine serum (FBS), penicillin streptomycin (PS), and Hank’s Balanced Salt Solution (HBSS) without calcium and magnesium were purchased from Thermo Fisher Scientific. HEPES, TritonX, bovine serum albumin (BSA), Tween20, and DAPI were purchased from Sigma Aldrich. Paraformaldehyde (PFA) and glass coverslips were purchased from Electron Microscopy Sciences. EDTA blood collection tubes were purchased from BD and Ficoll Paque was obtained from Cytiva. Human Pan T cell isolation kit was purchased from Miltenyi Biotech and Dynabeads^TM^ Human T-Activator CD3/CD28 was purchased from Thermo Fisher Scientific. Pre-conjugated flow cytometry antibodies, directed against phospho-NF-κB p65 (PE; cat. 12-9863-42), phospho-ZAP70/Sky Tyr319/Tyr352 (PE-Cyanine7; cat. 25-9006-42), CD25 (APC; cat. 17-0257-42), CD4 (Alexa Fluor^TM^ 700; cat. 56-0049-42), IFN-γ (PE; cat. 12-7319-41), TNF-α (PE; cat. 12-7349-41), and IL-2 (PE; cat. 12-7029-41) were purchased from Thermo Fisher Scientific. Additionally, anti-CD69 antibody (FITC; cat. 555530) was purchased from BD and GolgiPlug Protein Transport Inhibitor (cat. BDB555029) for intracellular cytokine production was purchased from Fisher Scientific. Monoclonal antibody directed against Piezo1 (Alexa Fluor^TM^ 488; cat. NBP1-78446AF488) and corresponding Rabbit IgG isotype control (Alexa Fluor^TM^ 488; cat. NBP2-24982) were purchased from Novus Biologicals. For confocal imaging, 10% normal goat serum (cat. 50-062Z), ActinRed^TM^555 ReadyProbes^TM^ reagent (cat. R37112), Alexa Fluor^TM^ 488 goat anti-rabbit IgG (H + L) (cat. A-11008), and phospho-NF-κB P65 (Ser536) monoclonal antibody (cat. MA5-15160) were purchased from Thermo Fisher Scientific and anti-fade mounting media (cat. H-1000-10) was purchased from Vectashield. Furthermore, cytospin funnels (cat. 10-354) were purchased from Fisher Scientific and a Super Pap hydrophobic pen (cat. 71312) was purchased from Electron Microscopy Sciences.

### Cell culture

Primary human T cells were cultured in RPMI-1640 medium containing 2 mM L-Glutamine, 25 mM HEPES, 10% FBS, and 1% PS. Cultures were maintained at a density of ∼500,000 cells/mL and incubated at 37 °C and 5% CO_2_. Freshly isolated T cells were allowed at least 4 h to recover and equilibrate in complete media prior to FSS exposure. Viability was confirmed to be ≥90% by trypan blue exclusion before initiation of experiments. For all experiments, individual patients’ T cells were selected based on overall cell health and total yield to ensure sufficient cells for each experimental timepoint.

### Human subject details

Whole blood samples were collected from prostate cancer patients who provided written informed consent prior to study enrollment and sample collection. All experimental work involving human subjects was performed in accordance with the Vanderbilt University Institutional Review Board (IRB) guidelines under an approved IRB protocol (Protocol no. 210748). Patient information collected included age at diagnosis, age and prostate specific antigen (PSA) serum levels at the time of sample collection, self-reported race and ethnicity, treatment history, Gleason score, and anatomical sites of metastasis. All patient samples and information remained deidentified for the entirety of the study.

Leukocyte-reduced whole blood units from healthy male donors were obtained from the Gulf Coast Blood Center in Houston, Texas. Healthy donor units were screened and confirmed negative for transfusion-transmissible infections, including human immunodeficiency virus (HIV), hepatitis B virus (HBV), hepatitis C virus (HCV), human T-lymphotropic virus (HTLV), and cytomegalovirus (CMV), West Nile Virus, Chagas disease, and syphilis. Donors included elderly male individuals to approximate the age distribution of the mCRPC patient cohort. Available healthy donor metadata included age, sex, self-reported race and ethnicity, and glycated hemoglobin (HbA1c), with all donors exhibiting normal glycemic status (HbA1c < 5.7%). All healthy donor samples were deidentified prior to transfer to the King Lab.

### PBMC isolation and processing

Fresh peripheral blood samples were obtained from patients undergoing laboratory testing at the Vanderbilt-Ingram Cancer Center (VICC) in Nashville, Tennessee, USA. Blood was collected in EDTA collection tubes and processed within 24 hours (h). For samples not processed immediately, collection tubes were placed on a tube rotator at 4 °C to preserve cell viability. Peripheral blood mononuclear cells (PBMCs) were isolated using Ficoll–Paque density gradient centrifugation and used immediately for downstream experiments.

PBMCs from healthy donor leukocyte-reduced blood units were isolated using an identical Ficoll–Paque density gradient protocol. Following isolation, healthy donor PBMCs were cryopreserved (90% FBS, 10% DMSO) and stored in liquid nitrogen (vapor phase) for less than 1 yr prior to use. For experiments, cryopreserved healthy donor PBMCs were rapidly thawed at 37 °C and immediately diluted in pre-warmed culture media and centrifuged at 200× *g* for 7 min. To reduce cell aggregation post-thaw, cells were incubated with DNaseI (50 U/mL) in HBSS (containing Ca^2+^/Mg^2+^) for 10 min at room temperature (RT). Cells were subsequently centrifuged at 200× *g* for 7 min and resuspended in appropriate T cell isolation buffer.

### Primary T cell isolation and activation

T cells were isolated by negative selection using the Human Pan T Cell isolation kit from Miltenyi Biotech, with samples passed through a 30 μm filter immediately prior to magnetic separation. For biochemical activation of T cells, Dynabeads^®^ Human T-Activator CD3/CD28 magnetic beads (herein referred to as αCD3/CD28) were added to the T cell culture according to the manufacturer’s instructions with a bead-to-cell ratio of 1:1. For T cells stimulated with both αCD3/CD28 and FSS, Dynabeads were added to culture media, gently mixed, and incubated at 37 °C and 5% CO_2_ for 15 min before FSS exposure to allow initial TCR-bead crosslinking to occur. T cells were loaded into cone-and-plate viscometers (Brookfield) and subjected to 5 dyn/cm^2^ for 1 h. The 4 experimental conditions, consisting of static, shear, static  +  αCD3/CD28, and shear  +  αCD3/CD28, were all subjected to the same experimental procedure to replicate identical conditions. For example, static conditions were loaded into viscometers but the rotor to impose FSS was not switched on. Immediately following the 1 h of FSS activation, static and shear samples were collected and cultured in fresh media. Importantly, bead-bound αCD3/CD28 was maintained in the culture media during and after FSS exposure for the remaining duration of experiments. The methodology used here for FSS activation of immune cells via cone-and-plate viscometer has been described extensively in previous work.^13^^,^^14^^,^^16^

### Flow cytometry sample preparation and staining

At the indicated timepoints, T cells were collected from culture, washed with HBSS, then fixed in 4% PFA for 10 min at RT. Samples were washed with HBSS before permeabilizing in 100% methanol on ice for 10 min. After permeabilization, cells were washed again in HBSS and samples were resuspended in 1% BSA and stored at 4 °C in the dark until antibody staining. For experiments quantifying intracellular cytokine production, GolgiPlug^TM^ Protein Transport Inhibitor was added to the T cell culture media 4 h prior to fixation and permeabilization. For Piezo1 expression, freshly isolated T cells were fixed and permeabilized before staining with antibody targeting Piezo1 and its corresponding isotype control (prepared in triplicate). All antibody staining was performed in 1% BSA containing fluorochrome-conjugated antibodies and incubated at RT for 15 min in the dark.

Flow cytometry experiments measuring NF-κB phosphorylation, intracellular cytokine production, and Piezo1 expression were acquired in a Guava easyCyte 12HT flow cytometer (Cytek Biosciences); analysis was performed in FlowJo software (BD). Flow cytometry experiments quantifying ZAP70 phosphorylation, CD69, CD25, CD4, and CD45Ro were acquired in a Novocyte Quanteon flow cytometer (Agilent) and analysis was performed in the NovoExpress software (Agilent).

### Confocal microscopy

At 72 h post-activation, T cells were collected from culture and washed 3 times in HBSS. Cells were fixed in 4% PFA for 15 min at RT, then washed again in HBSS. Samples were resuspended in 1% BSA and cytospun onto a glass slide using the Thermo Shandon Cytospin 3 at 1,000 RPM for 4 min. Slides were removed from the cytospin, and the cells confined by drawing a circle around the cell deposition area with a hydrophobic pen. Slides were set to dry at RT for at least 1 h. All microscopy slide washes consisted of three washes in HBSS with 0.02% Tween20 for 5 min per wash. Samples were permeabilized using 1X Triton for 10 min, washed, then blocked in 5% BSA and 5% normal goat serum for 2 h at RT. Antibody solutions were prepared fresh in blocking solution at each use. Primary antibody targeting phosphorylated NF-κB was added to the cell samples (1:100) and incubated overnight at 4 °C. After primary antibody incubation, samples were washed, then incubated in blocking solution containing Alexa Fluor^TM^ 488 secondary antibody (1:200), ActinRed^TM^555 ReadyProbes^TM^ reagent (1 drop:1,000), and DAPI (1:1,000) for 45 min at RT in the absence of light. Samples were washed for a final time and 18 mm glass coverslips were mounted over the cell deposition area using Vectashield anti-fade mounting media and sealed with clear nail polish.

Confocal images were acquired on a Zeiss LSM900 using the 63X oil-immersion objective. Image acquisition settings were saved and reused in the Zeiss ZEN software for each experiment to ensure identical settings were used between replicates. Cell fluorescence quantification was carried out using an image processing algorithm in MATLAB, described extensively in previous work.^13^ Fluorescence signal per cell is reported as the corrected total cell fluorescence (CTCF), which is calculated in the MATLAB program as:


(1) $$\mathrm{CTCF}={\mathrm{IntDensity}}_{\mathrm{cell}}-({\mathrm{Area}}_{\mathrm{cell}}\times {\mathrm{Mean}}_{\mathrm{bkg}}),\;$$

where *IntDensity_cell_* is the integrated density of the cell fluorescence, *Area_cell_* is the area of the cell, and Mean_bkg_ is the mean fluorescence intensity of the background. Representative confocal images were rendered in the FIJI software, where brightness and contrast were adjusted linearly across the entire image to facilitate viewing.

### Quantification of synergy using Jin’s formula

To evaluate the effect of combined FSS and αCD3/CD28 on T cell activation, Jin’s Q-value formula was used to quantify synergy.^19^ The Q-value is defined as:


(2) $$Q=\frac{E_{a+b}}{E_{a}+E_{b}-(E_{a}\times E_{b})},$$

where *E_a_*, *E_b_*, and *E_a+b_* represent the normalized activation level achieved from only FSS, only αCD3/CD28, and the combination of FSS and αCD3/CD28 stimulation, respectively. For each donor and timepoint, activation level was determined by the median fluorescence intensity (MFI) of phosphorylated NF-κB normalized to the static control condition. To account for the inter-donor variability in activation magnitude, data for each donor at each timepoint was scaled using the equation:


(3) $$E=\frac{{\mathrm{MFI}}_{\mathrm{Condition}}-{\mathrm{MFI}}_{\mathrm{Static}}}{{\mathrm{MFI}}_{\mathrm{Max}}-{\mathrm{MFI}}_{\mathrm{Static}}},$$

where *MFI_Condition_* represents the normalized MFI for a given experimental condition (FSS, αCD3/CD28, or FSS + αCD3/CD28), *MFI_Max_* represents the highest normalized MFI value observed among all 4 conditions for that donor, and *MFI_Static_* refers to the normalized MFI value of the static control, which in this case, equates to 1 for all donors. This calculates an “effect fraction” that constrains *E* between 0 (no activation or activation below the baseline) and 1 (maximal activation), representing the rise in activation above the baseline static condition, scaled to the donor’s full activation range and allowing direct comparison across donors. Consistent with standard practice, when *E *< 0, *E* is set equal to zero, and when *E *> 1, *E* is set equal to 1. *Q* values [dimensionless] were calculated independently for each donor and timepoint, where *Q *< 0.85 indicates antagonism, 0.85 ≤ *Q *≥ 1.15 indicates an additive effect, and *Q *> 1.15 indicates synergy between FSS and αCD3/CD28 stimulation.

### Quantification of the area under the curve

To quantify the overall magnitude and duration of activation from FSS and αCD3/CD28 stimulation, the area under the curve (AUC) was determined using NF-κB phosphorylation data (FSS, αCD3/CD28, and FSS + αCD3/CD28 stimulation) and Jin’s *Q* values over time (0, 24, 28, 72 h). The AUC was calculated using the trapezoidal rule, which is a standard approach to integrating biological data over discrete, uniformly spaced timepoints:


$$\mathrm{AUC}=\;\sum_{i=1}^{n-1}\frac{\left(X_{i}+X_{i+1}\right)}{2}\;\times \;(t_{i+1}-t_{i})$$

where *X_i_* and *X_i+1_* represent NF-κB phosphorylation (MFI normalized to static) and Jin’s Q-values at respective timepoints *t_i_* and *t_i+1_*. Since this equation gives AUC in units of [h], it was then divided by 72 h to obtain a normalized AUC [dimensionless] that represents the degree of activation or synergy over time, which can be compared across donors.

### Principal component analysis

Principal component analysis (PCA) was performed to identify the main sources of variation observed between patient-derived T cell response to activation over time. The input dataset consisted of the z-score standardized Jin’s *Q* value AUC and NF-κB phosphorylation AUC from FSS, αCD3/CD28, and FSS + αCD3/CD28 stimulation) FSS stimulation. AUC values were chosen rather than the mean or single timepoint values because AUC captures the full temporal profile of activation for each donor, accounting for differences observed in activation kinetics over time.

All AUC values were standardized (mean = 0, standard deviation = 1) and PCA was performed in Python using the scikit-learn package, “sklearn.decomposition.PCA.”^20^ Principal components, PC1 and PC2, were used as the primary axes, representing the main source of biological variation. Feature loading coefficients were quantified to determine the relative contribution of each stimulation condition to the total variance from PC1 and PC2. Final PCA scores for each patient were plotted in Graphpad Prism, where each donor point was color-coded based on Piezo1 expression (MFI normalized to isotype control).

### Statistical analysis

Statistical analysis and graph creation was performed in GraphPad Prism. All data presented in bar graphs represent the mean ± SEM, and each point within a condition represents an independent experiment using T cells obtained from a unique donor. Unless otherwise stated, the repeated measure analysis of variance (RM-ANOVA) statistical test was performed with Tukey’s multiple comparison post-hoc test for statistical significance (α = 0.05). For confocal microscopy data, boxplots express the median (central line), first and third quartiles (bounds of box), and the highest and lowest values (whiskers), where each dot represents one individual cell from *N* = 3 unique patients. Statistics for confocal microscopy data were calculated by an ordinary one-way ANOVA with Tukey’s multiple comparison post hoc test (α = 0.05). Statistical significance between conditions is denoted as: *P < 0.05, **P < 0.01, ***P < 0.001, ****P < 0.0001.

## Results

### Study population

The study population was comprised of 8 men with mCRPC (Table 1). Mean age in years (yrs) at diagnosis was 66.1 yrs (range: 60–74 yrs) and mean age at sample collection was 73.6 yr (range: 63–84 yr). Including the treatment at the time of sample collection, subjects had a range of therapies from 1–7 (median: 5). Mean PSA at the time of sample collection was 248.84 ng/mL (range: 0.1–1,349.79 ng/mL). To provide a baseline reference for T cell responses, a cohort of healthy male donors (*n* = 3) was included for comparison. Healthy donors were selected to approximate the age distribution of the mCRPC cohort. Individual donors are referred to throughout the manuscript as M1 (62 yrs, Caucasian), M2 (69 yr, race=Other), and M3 (73 yr, Caucasian).

**Table 1:** Deidentified patient information for the 8 mCRPC patients who participated in this study.

Patient	K6	K10	K14	K15	K16	K17	K20	K23
Race	CA	CA	CA	CA	CA	CA	CA	CA
Ethnicity	NH	NH	NH	NH	NR	NH	NH	H
Age at diagnosis	61	62	65	74	73	60	64	70
Age at time of sample collection	73	63	69	84	77	78	69	76
PSA at time of collection (ng/mL)	9.59	< 0.10**^b^**	9.55	21.17	1,349.79	58.38	542.03	< 0.10**^b^**
PSA50 reached^c^	Y	Y	N	N	N	Y	N	NA
Radiographic response^c^	Y	Y	N	N	N	UNK	N	Y
Gleason score	NR	NR	5 + 4 = 9	3 + 4 = 7	4 + 5 = 9	3 + 4 = 7	5 + 4 = 9	4 + 5 = 9
Metastatic lesions								
* Bone*	X	X		X	X	X	X	X
* Lymph node*	X	X	X			X	X	
* Lung*	X							X
* Liver*					X	X	X	
Treatment at time of collection^a^	^177^Lu (Pre-C5)	Abiraterone & prednisone	^177^Lu (Pre-C2)	^177^Lu (Pre-C2)	^177^Lu (Pre-C1)	^177^Lu (Pre-C3)	^177^Lu (Pre-C3)	^177^Lu (Pre-C1)
Prior treatments								
* Bicalutamide*		X			X	X		
* Abiraterone*	X		X	X	X	X		X
* Apalutamide*	X						X	
* Enzalutamide*	X		X	X	X	X	X	X
* Docetaxel*	X		X	X		X	X	X
* Cabazitaxel*	X					X	X	
* Radium-223*					X			
* PARP inhibitor*	X		X					
* Sipuleucel-T*			X	X				

a ^177^Lu: Lutetium Vipivotide Tetraxetan is administered in 6 cycles. Samples were collected immediately before treatment cycle 1–6, denoted as Pre-C#.

b Patient K23 has non-PSA expressing prostate cancer. Therefore, PSA50 is NA; Patient K10 did experience 50% decline in PSA on the treatment at time of sample collection.

c PSA50 and radiographic response refer to patient response on the treatment at time of sample collection.

Note. Y, Yes; N, No; NA, Not applicable; UNK, Unknown; NR, Not released; CA, Caucasian; NH, Not Hispanic; H, Hispanic.

### Fluid shear stress stimulation enhances NF-κB phosphorylation in patient-derived T cells independently and synergistically with αCD3/CD28 stimulation

To determine how FSS influences the activation signaling of human T cells over time, NF-κB phosphorylation was quantified following mechanical stimulation in T cells isolated from mCRPC patients and age-matched healthy male donors. Pan-T cells were isolated from PBMCs using magnetic separation (negative selection) and were exposed to constant and uniform FSS (5 dyn/cm^2^) in cone-and-plate viscometers for 1 h in the presence (stimulated) and absence (unstimulated) of biochemical activation via TCR-crosslinking antibody conjugated beads (αCD3/CD28). The shear magnitude and exposure duration were selected based on a prior dose- and time-response study demonstrating graded increases in proximal TCR signaling within this range, which also corresponds to physiologically relevant wall shear stresses observed in circulation.^12^

**Figure 1:**
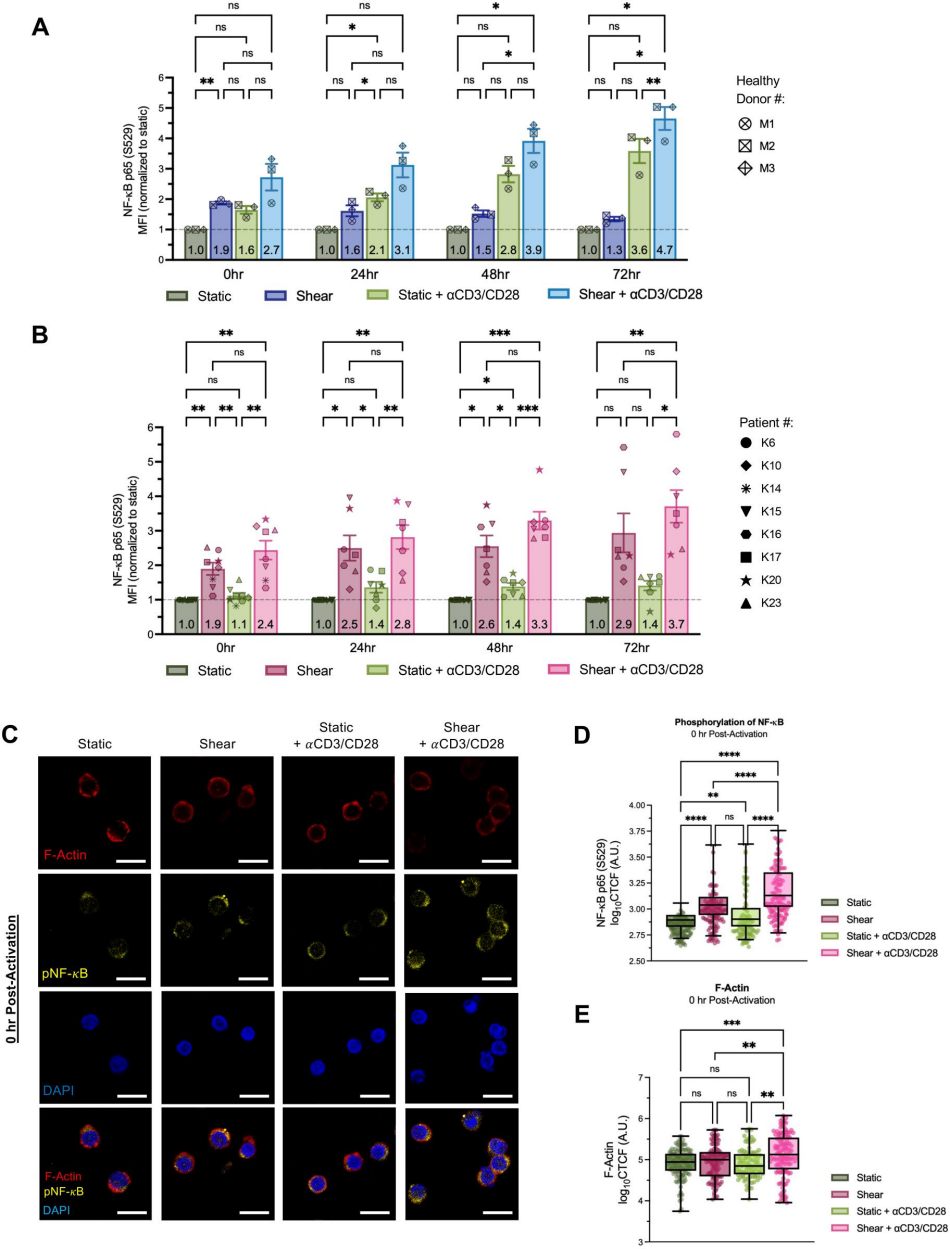
Fluid shear stress exposure enhances NF-κB phosphorylation in patient-derived T cells independently and synergistically with biochemical activation. (A, B) Median fluorescence intensity (MFI) of NF-κB phosphorylation (normalized to static) over 72 h post-activation in flow cytometry in T cells derived from (A) healthy male donors (*n* = 3) and (B) mCRPC patients (0 h: *n* = 8, 24–72 h: *n* = 7). (C-E) Representative (C) confocal images and quantification of (D) NF-κB phosphorylation and (E) F-actin immediately following activation in mCRPC patient-derived T cells (pNF-κB = yellow; F-actin = red; DAPI = blue; scale bar = 10μm; *n* = 3 unique donors). (A, B) Data are shown as mean ± SEM with individual donor values overlaid and statistics calculated by repeated-measures two-way ANOVA and Tukey’s multiple comparisons post hoc test. (D, E) Boxplots represent the median (central line), first and third quartiles (bounds of box), and the highest and lowest values (whiskers), with points representing analyzed cells overlaid. Statistics calculated using ordinary one-way ANOVA with Tukey’s multiple comparisons test. No significance (ns), *P < 0.05, **P < 0.01, ***P < 0.001, ****P < 0.0001.

Phosphorylation of the transcription factor NF-κB (p65 subunit, Ser529; pNF-κB) was quantified by flow cytometry in T cells isolated from elderly healthy male donors as median fluorescence intensity (MFI) over a 72 h period (Fig. 1A). Immediately following FSS exposure (0 h), healthy donor T cells exposed to FSS alone exhibited a significant increase in pNF-κB (1.9-fold change relative to static, *P* = 0.0084), whereas αCD3/CD28 alone led to a smaller increase (1.6-fold, *P* = 0.0904). The combination of αCD3/CD28 and FSS produced the greatest pNF-κB activation (2.7-fold, *P* = 0.5112). Across later timepoints, T cells exposed to only FSS gradually decreased in pNF-κB over time, with a 1.6-, 1.5-, and 1.3-fold change relative to static at 24 h, 48 h, and 72 h, respectively. In contrast, T cells stimulated with αCD3/CD28 in both the presence and absence of FSS continued to increase in pNF-κB across later timepoints, consistent with sustained TCR-mediated activation signaling. Stimulation with only αCD3/CD28 increased pNF-κB by 2.1-, 2.8, and 3.6-fold relative to static at 24 h, 48 h, and 72 h, respectively. Notably, when FSS was combined with αCD3/CD28 stimulation, pNF-κB steadily increased over 72 h and exhibited the greatest pNF-κB levels across conditions (3.1-, 3.9-, 4.7-fold, at 24 h, 48 h, and 72 h respectively). These data indicate that mechanical stimulation enhances TCR-mediated activation signaling over time in elderly healthy male T cells, consistent with our previous studies utilizing immortalized and healthy donor (<40 yrs) T cells.

Having established that FSS enhances NF-κB phosphorylation in healthy donor T cells, we next investigated whether similar responses occur in T cells isolated from mCRPC patients, where each unique donor is denoted as “K#” (Table 1). Isolated pan-T cells were exposed to identical stimulation conditions as described above and pNF-κB was quantified over 72 h (Fig. 1B and S1). Consistent with healthy donor T cells, mCRPC T cells exposed to FSS alone displayed a significant increase in NF-κB phosphorylation immediately following FSS exposure (1.9-fold relative to static, *P* = 0.0053). However, in contrast to healthy donor T cells, mCRPC T cells exhibited a markedly weaker response to αCD3/CD28 stimulation alone, with only a modest and non-significant increase in pNF-κB at 0 h (1.1-fold, *P* = 0.5112). Importantly, the combination of FSS with αCD3/CD28 stimulation resulted in the highest levels of NF-κB phosphorylation (2.4-fold, *P* = 0.0212), suggesting that mechanical stimulation can enhance otherwise limited TCR-mediated activation signaling in patient-derived T cells.

NF-κB phosphorylation in mCRPC T cells progressively increased over time in all stimulation conditions. While FSS alone produced a sustained increase in pNF-κB over time, reaching a 2.9-fold increase relative to static at 72 h (*P* = 0.0519), αCD3/CD28 alone led to only a modest increase in pNF-κB (1.4-fold, *P* = 0.0922). In contrast, the combination of FSS and αCD3/CD28 stimulation resulted in the greatest pNF-κB levels, resulting in a 3.7-fold increase relative to static at 72 h (*P* = 0.0049). Moreover, the combined FSS and αCD3/CD28 stimulation condition consistently demonstrated significantly higher NF-κB phosphorylation compared to the static conditions without (*P* < 0.01) and with αCD3/CD28 stimulation (*P* < 0.05) at every measured timepoint (Fig. 1B). Notably, FSS alone maintained higher NF-κB phosphorylation relative to both static unstimulated and static αCD3/CD28 stimulated T cells, supporting the idea that FSS can independently induce NF-κB activation signaling. While patient-to-patient variability was observed across the cohort, the overall trend of increased NF-κB phosphorylation in both FSS conditions remained consistent and statistically significant compared to static conditions. These results suggest that FSS can induce activation signaling in the presence and absence of αCD3/CD28 stimulation in mCRPC T cells, despite having reduced responsiveness to TCR-stimulation alone.

Immunofluorescence confocal microscopy was performed on patient-derived T cells 72 h post-activation to quantify pNF-κB and F-actin (Fig. 1C). Since the actin cytoskeleton integrates mechanical and TCR-derived signals and can be modulated by FSS, F-actin was evaluated to determine whether changes in pNF-κB signaling were accompanied by overall cytoskeletal actin content. An automated image analysis algorithm in MATLAB was used to quantify fluorescence intensity using the CTCF.^13^ Consistent with flow cytometry data, FSS caused a moderate increase in pNF-κB fluorescence intensity compared to static controls, whereas combined FSS and αCD3/CD28 stimulation resulted in the highest pNF-κB signal compared to all other conditions (Fig. 1D). The statistical significance between the shear and the shear with αCD3/CD28 stimulation conditions at 72 h are the only discrepancy between the flow cytometry (*P* = 0.4271) and microscopy (*P* < 0.0001) analysis. This discrepancy is likely driven by intrinsic variability in T cell responsiveness within the smaller subset of patient-derived cells analyzed by microscopy (K16, K17, K20), where differences in both mechanosensitivity and αCD3/CD28 sensitivity may have led to a more pronounced activation response in the combined stimulation conditions. In addition, microscopy analyses revealed that combined FSS and αCD3/CD28 led to a statistically significant increase in total F-actin CTCF compared to all other conditions (Static: *P* = 0.0007; Shear: *P* = 0.0089; Static + αCD3/CD28 = 0.0014). Moreover, F-actin signal was not reduced under shear exposure relative to static conditions (*P* < 0.05), indicating that enhanced pNF-κB activation was not associated with a loss of overall actin content (Fig. 1E). Taken together, these findings are consistent with activation-associated actin remodeling occurring alongside enhanced pNF-κB signaling, rather than nonspecific cytoskeletal disruption.

### Combined mechanical and biochemical stimulation enhances cytokine production and activation marker expression in patient-derived T cells

To determine how mechanical forces influence downstream activation and effector function, T cells isolated from mCRPC patients and age-matched healthy male donors were stimulated with αCD3/CD28 beads under static or shear conditions for 72 h, and intracellular cytokine production and activation marker expression were assessed by flow cytometry. IFN-γ, TNF-α, and IL-2 were selected because they represent key effector (IFN-γ, TNF-α) and autocrine (IL-2) cytokines that are directly downstream of NF-κB and canonical TCR signaling pathways. The degree of synergy between FSS and αCD3/CD28 was quantified according to Jin’s formula, where the calculated Q-value corresponds with an antagonistic (Q < 0.85), additive (0.85 ≤ Q ≥ 1.15), or synergistic (Q > 1.15) relationship (Fig. S2).

**Figure 2:**
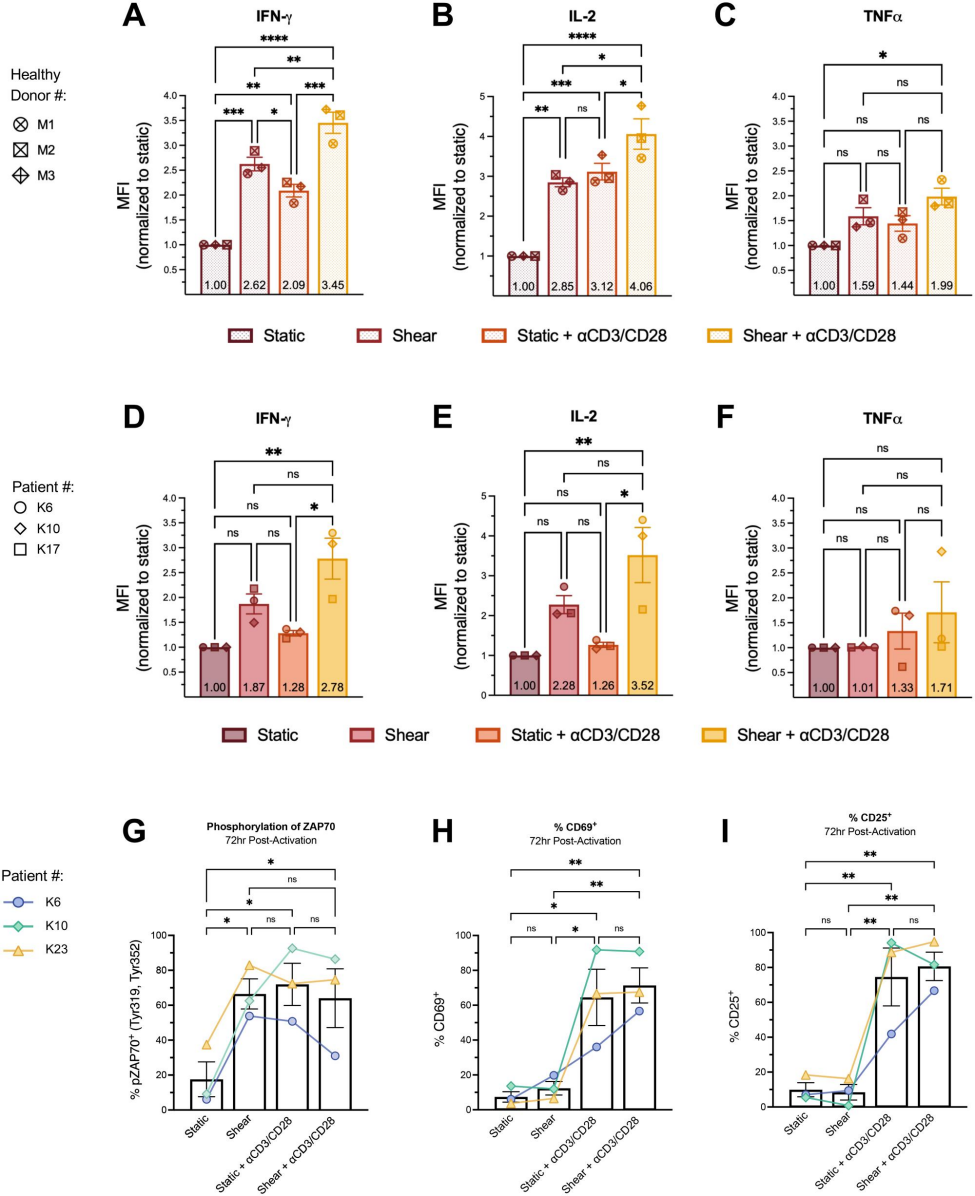
Combined mechanical and αCD3/CD28 stimulation enhances cytokine production and activation marker expression in patient-derived T cells. (A–C) Intracellular cytokine production of (A) IFN-γ, (B) IL-2, and (C) TNF-α in T cells from unique elderly healthy male donors (n = 3), presented as MFI normalized to static control. (D–F) Intracellular cytokine production of (D) IFN-γ, (E) IL-2, and (F) TNF-α in T cells from unique mCRPC patients (*n* = 3), presented as MFI normalized to static control. (G–I) Quantification of (G) ZAP70 (Tyr319/Tyr352) phosphorylation, (H) CD69 expression and (I) CD25 expression 72 h post-activation in flow cytometry. Data are shown as mean ± SEM with individual donor values overlaid. Statistics calculated by repeated-measures one-way ANOVA with Tukey’s multiple comparisons test. No significance (ns), *P < 0.05, **P < 0.01, ***P < 0.001, ****P < 0.0001.

In T cells isolated from older healthy male donors, both mechanical and TCR-mediated stimulation increased cytokine production relative to the unstimulated static control (Fig. 2A–C, S2A). For IFN-γ production (Fig. 2A), FSS alone induced a robust increase (2.62-fold relative to static) that exceeded the response to αCD3/CD28 stimulation alone (2.09-fold), while combined stimulation synergistically increased IFN-γ production 3.45-fold (*Q* = 1.23). IL-2 production (Fig. 2B) increased substantially with both FSS (2.85-fold) and αCD3/CD28 stimulation (3.12-fold), with the greatest increase observed when combined (4.06-fold), consistent with an additive interaction that approaches the threshold for synergy (*Q* = 1.13). In contrast, TNF-α production (Fig. 2C) showed more modest changes across stimulation conditions, with observed increases following FSS alone (1.59-fold) and αCD3/CD28 alone (1.44-fold) and similarly reached its highest level with the combination of FSS and αCD3/CD28 (1.99-fold; *Q *= 1.51). Taken together, these results demonstrate that mechanical stimulation enhances cytokine production in healthy donor T cells and cooperates with TCR signaling to amplify both effector and autocrine activation pathways.

Cytokine production was next evaluated in T cells isolated from mCRPC patients (Fig. 2D–F). Compared to healthy donors, patient-derived T cells exhibited reduced responsiveness to αCD3/CD28 stimulation alone, with minimal increases in IFN-γ (Fig. 2D) and IL-2 (Fig. 2E) production by 1.28- and 1.26-fold relative to static, respectively (*P* > 0.8). In contrast, FSS alone modestly enhanced cytokine production, increasing IFN-γ and IL-2 by 1.87-fold (*P* = 0.1530) and 2.28-fold (*P* = 0.1247), respectively, relative to static conditions. Although these increases from FSS stimulation alone were not statistically significant, the trend agrees with healthy T cell data and suggests that mechanical stimulation may independently promote cytokine synthesis in the absence of biochemical activation. Moreover, when FSS was combined with αCD3/CD28 stimulation, IFN-γ and IL-2 production was further increased from the static condition by 2.78- (*P* = 0.0084) and 3.52-fold (*P* = 0.0072), respectively, demonstrating synergistic activation of both effector (IFN-γ; *Q* = 1.89) and autocrine (IL-2; *Q* = 1.82) signaling pathways.

In contrast to IFN-γ and IL-2, TNF-α production exhibited substantial patient-to-patient variability under both static and shear conditions (Fig. 2F). When αCD3/CD28 stimulation was combined with FSS, an increase in TNF-α was observed in one patient (K10: 2.83-fold relative to static), with little to no effect in a second (K6: 1.18-fold relative to static) and third (K17: 1.02-fold relative to static) patient. Across all donors, the average *Q* value (*Q* = 2.63) indicated synergy between FSS and αCD3/CD28, however, this effect was largely driven by inter-patient variability rather than a consistent synergistic response from all donors. High variability was also observed in T cells stimulated with only αCD3/CD28, further indicating that TNF-α production is intrinsically heterogeneous among donors and not exclusively dependent on mechanical stimulation.

In parallel, proximal TCR signaling and downstream activation marker expression were evaluated in both mCRPC patient-derived T cells and elderly healthy donor T cells. In mCRPC T cells, FSS alone significantly increased the phosphorylation of ZAP70 (Tyr319/Tyr352) compared to the static condition (Static: 17.55%, Shear: 66.45%, *P* = 0.0312), reaching comparable levels to αCD3/CD28 stimulation alone (Fig. 2G). In contrast, healthy donor T cells exhibited minimal ZAP70 phosphorylation with FSS alone, with statistically significant increases observed only in the conditions stimulated with αCD3/CD28 (Fig. S3A). These findings indicate that mechanical stimulation can independently activate proximal TCR signaling in mCRPC T cells to a greater extent than in healthy donor cells.

T cell activation surface markers, CD69 (Fig. 2H and S3B) and CD25 (Fig. 2I and S3C), were also evaluated in flow cytometry at 72 h post-activation. Static and shear conditions without αCD3/CD28 stimulation retained low CD69 (Static: 7.8%, Shear: 12.7%) and CD25 (Static: 10.3%, Shear: 8.8%) expression, where αCD3/CD28 stimulation in both static and shear conditions resulted in robust upregulation of CD69 (Static + αCD3/CD28: 64.8%; Shear  + αCD3/CD28: 71.7%) and CD25 (Static + αCD3/CD28: 74.9%; Shear  + αCD3/CD28: 81.0%). These trends were similarly observed in T cells isolated from elderly healthy male donors (Fig. S3B, C). Average Q-values for CD69 (*Q* = 1.14) and CD25 (*Q* = 1.20) in mCRPC T cells demonstrated an additive and mildly synergistic relationship between FSS and αCD3/CD28 stimulation, respectively, with both values lying near the defined synergy threshold of *Q* > 1.15 (Fig. S2B). While the overall trends were consistent, patient-dependent variability was observed in the overall magnitude of activation. For example, patients K10 and K23 showed higher expression of CD69 and CD25 under both static and shear αCD3/CD28 stimulation, whereas K6 showed a comparatively lower response overall. Importantly, although K6 had lower overall surface receptor expression, there was a notable increase observed in both CD69 and CD25 when αCD3/CD28 stimulation was combined FSS, compared to αCD3/CD28 alone. Taken together, these findings indicate that biochemical activation through αCD3/CD28 is the primary driver of surface activation marker expression in healthy and patient-derived T cells.

### Fluid shear stress differentially modulates CD4^+^ T cell activation and memory acquisition

While CD8^+^ T cells are traditionally viewed as the primary effectors of antitumor immunity, CD4^+^ T cells have been increasingly recognized as major determinants of the success of immunotherapy.^21^^,^^22^ CD4^+^ T cells exhibit clearly defined surface marker transitions during activation and differentiation, making them an informative subset for evaluating FSS-mediated effects. Investigating memory phenotypes in CD4^+^ T cells is particularly relevant, as durable CD4^+^ memory acquisition and persistence has been associated with improved therapeutic outcomes in cancer patients.^23^

**Figure 3:**
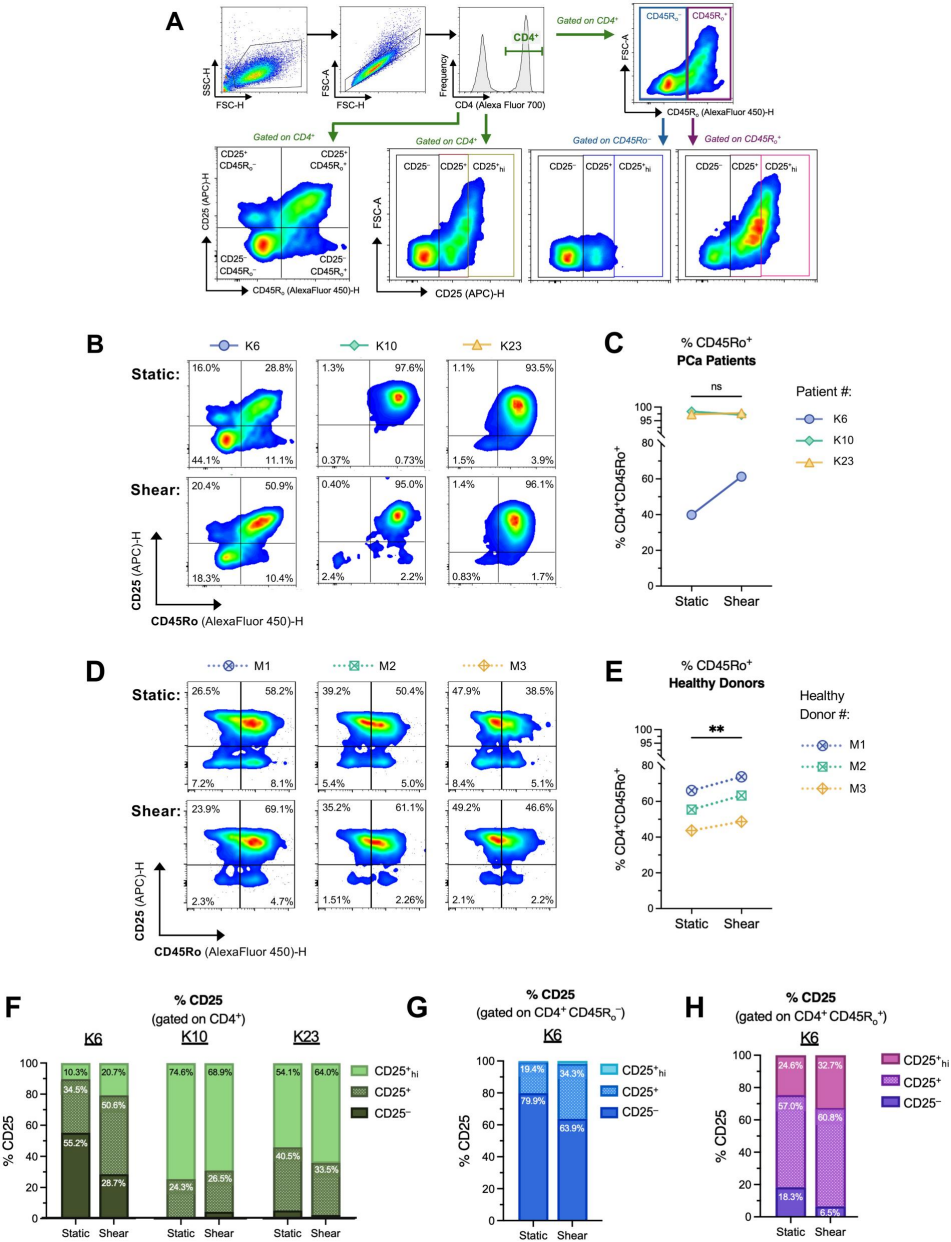
Fluid shear stress modulates CD4^+^ T cell memory acquisition and activation states in mCRPC patients and healthy male donors. (A) Representative flow cytometry gating strategy for identifying CD4^+^ T cells and CD25 expression across CD45Ro^−^ and CD45Ro^+^ subset following 72 h activation with αCD3/CD28 beads under static or shear conditions. (B-E) Representative flow cytometry density plots of CD45Ro vs. CD25 fluorescence in CD4^+^ T cells from (B) mCRPC patients and (D) healthy male donors under static and shear conditions, with corresponding quantification of CD45Ro^+^ frequency shown for (C) mCRPC patient-derived (*n*=3) and (E) healthy male donor T cells (*n*=3). Statistical comparisons were performed using paired ratio *t* tests (two-tailed; **P < 0.01). (F-H) Stacked bar graphs showing relative proportions of CD25^−^, CD25^+^, and CD$25_{\text{hi}}^{+}$ populations within (F) total CD4^+^ T cells, (G) CD45Ro^−^ T cells, and (H) CD45RO^+^ T cells.

To determine how FSS influences CD4^+^ T cell activation and memory acquisition, CD25^-^/CD25^+^/CD$25_{\text{hi}}^{+}$ and CD45Ro subsets were quantified in flow cytometry 72 h following αCD3/CD28 stimulation under static and shear conditions. Analyses were performed in T cells derived from three mCRPC patients (K6, K10, and K23) and healthy donors. Due to limited cell yields and varying growth kinetics in patient T cells, different donor sets were used for the cytokine and phenotypic analyses to ensure sufficient sample quantity for each assay. All results presented here reflect αCD3/CD28 stimulated conditions, as unstimulated T cells do not undergo TCR-dependent CD25 upregulation or CD45Ro conversion. Using flow cytometry gating (Fig. 3A), cell debris and cell clusters were excluded before further delineating CD4^+^ T cells. From the CD4^+^ population, CD45Ro^−^ (naive-like), CD45Ro^+^ (memory), and CD25^-^/CD25^+^/CD$25_{\text{hi}}^{+}$ gates were used to evaluate memory acquisition and CD25 activation states, respectively.

The baseline and FSS-induced distribution of CD45Ro phenotypes varied considerably between mCRPC patient (Fig. 3B) and healthy donor T cells (Fig. 3D). In patient-derived T cells, FSS exposure led to a pronounced shift toward memory phenotype in K6 (Static: 39.9%, Shear: 61.3%), indicating enhanced memory acquisition (Fig. 3C). In contrast, the CD4^+^ populations in K10 and K23 were already primarily CD45Ro^+^ under static conditions (>95%) and showed negligible, non-directional change with FSS exposure, suggesting a ceiling effect on memory acquisition at this timepoint. When reviewing the treatment history of patients, there was no clear trend which may explain these findings in regards to prior therapies (eg chemotherapy naive vs exposed, or prior treatment with sipuleucel-T immunotherapy). When pooled across the three patients, the effect of FSS was statistically non-significant (*P* = 0.4335), reflecting inter-patient heterogeneity from inherent baseline differences. However, the directional K6 response indicates that FSS may promote memory phenotype acquisition in at least a subset of patients with a larger CD45Ro^-^ (naive-like) basal population.

In elderly healthy male donor CD4^+^ T cells, FSS led to a minimal but consistent increase of CD45Ro^+^ cells across all donors (Fig. 3E), with the mean CD45Ro^+^ expression rising from 55.1% under static conditions to 61.9% with FSS (*P* = 0.0047). As the magnitude of this increase was modest and the sample size remains limited, these findings should be interpreted as exploratory. However, the consistent shift toward a memory phenotype in each donor supports the concept that FSS may promote CD45Ro acquisition in activated T cells. Notably, the magnitude and direction of the response observed in K6 more closely resembled the behavior of healthy donor T cells, whereas K10 and K23 were already maximally CD45Ro^+^ at baseline. Taken together, these findings suggest that FSS enhances memory-associated phenotypic transitions most effectively in T cells that are not already maximally differentiated, reinforcing a context-dependent role for mechanical stimulation in shaping CD4^+^ T cell fate.

CD25 (IL-2Rα) is rapidly induced by TCR stimulation, where the CD25^+^ fraction reports activation magnitude and CD$25_{\text{hi}}^{+}$ represents the subset with heightened IL-2 responsiveness and proliferative potential. In mCRPC-derived CD4^+^ T cells (Fig. 3F), substantial inter-patient variability was observed. K6 possessed a large CD25^-^ subset under static conditions, which was significantly decreased under FSS conditions (Static: 55.2%; Shear: 28.7%). A reciprocal enrichment of both CD25^+^ (Static: 34.5%; Shear: 50.6%) and CD$25_{\text{hi}}^{+}$ (Static: 10.3%; Shear: 20.7%) was observed in the sheared K6 T cells, indicating FSS-induced sustained activation. In contrast, K10 and K23 displayed predominantly CD25^+^/CD$25_{\text{hi}}^{+}$ phenotypes in both static and shear treatments, with less than 6% as CD25^-^ in all conditions. Notably, K23 showed an FSS-induced increase in the CD$25_{\text{hi}}^{+}$ subset (Static: 54.1%; Shear: 64.0%), whereas K10 exhibited a modest decrease (Static: 74.6%; Shear: 68.9%). In healthy donor CD4^+^ T cells, CD25 subset distributions were largely preserved between static and shear conditions, with no statistically significant differences observed (Fig. S4A). However, a consistent reduction in the CD25^-^ population was observed across all donors, accompanied by reciprocal redistribution between CD25^+^ and CD$25_{\text{hi}}^{+}$ subsets.

To further assess the impact of FSS on the activation status of memory CD4^+^ T cells, CD25^-^/CD25^+^/CD$25_{\text{hi}}^{+}$ subsets were evaluated in CD45Ro^-^ and CD45Ro^+^ populations. In mCRPC patient-derived T cells, analysis of the CD45Ro^-^ subset was only performed for K6 due to near absence of this population in K10 and K23 (<5%). Within K6 CD45Ro^-^ cells (Fig. 3G), FSS reduced CD25^-^ (Static: 79.9%; Shear: 63.9%) and increased CD25^+^ expression (Static: 19.4%; Shear: 34.3%), indicating FSS-induced priming of CD45Ro^-^ cells. Furthermore, less than 2% of K6 CD45Ro^-^ cells were CD$25_{\text{hi}}^{+}$, as expected within this population. Within the K6 CD45Ro^+^ memory cells (Fig. 3H), FSS increased both CD25^+^ (Static: 57.0%; Shear: 60.8%) and CD$25_{\text{hi}}^{+}$ (Static: 24.6%; Shear: 32.7%) populations, with a reciprocal decrease in CD25^-^ expression (Static: 18.3%; Shear: 6.5%), indicating enhanced FSS-induced activation of memory cells. A similar pattern was observed in healthy donor T cells, where FSS consistently reduced the CD25^-^ population across both CD45Ro^-^ and CD45Ro^+^ subsets, accompanied by redistribution into CD25^+^ and CD25^+^hi states (Fig. S4B, C). Although the magnitude of these shifts was modest and did not reach statistical significance, the directionality was consistent across all donors, reflecting subtle refinement of activation states in an already robustly activated population.

### Synergy quantification and principal component analysis reveal donor-dependent mechanical and antigen-driven activation profiles

**Figure 4:**
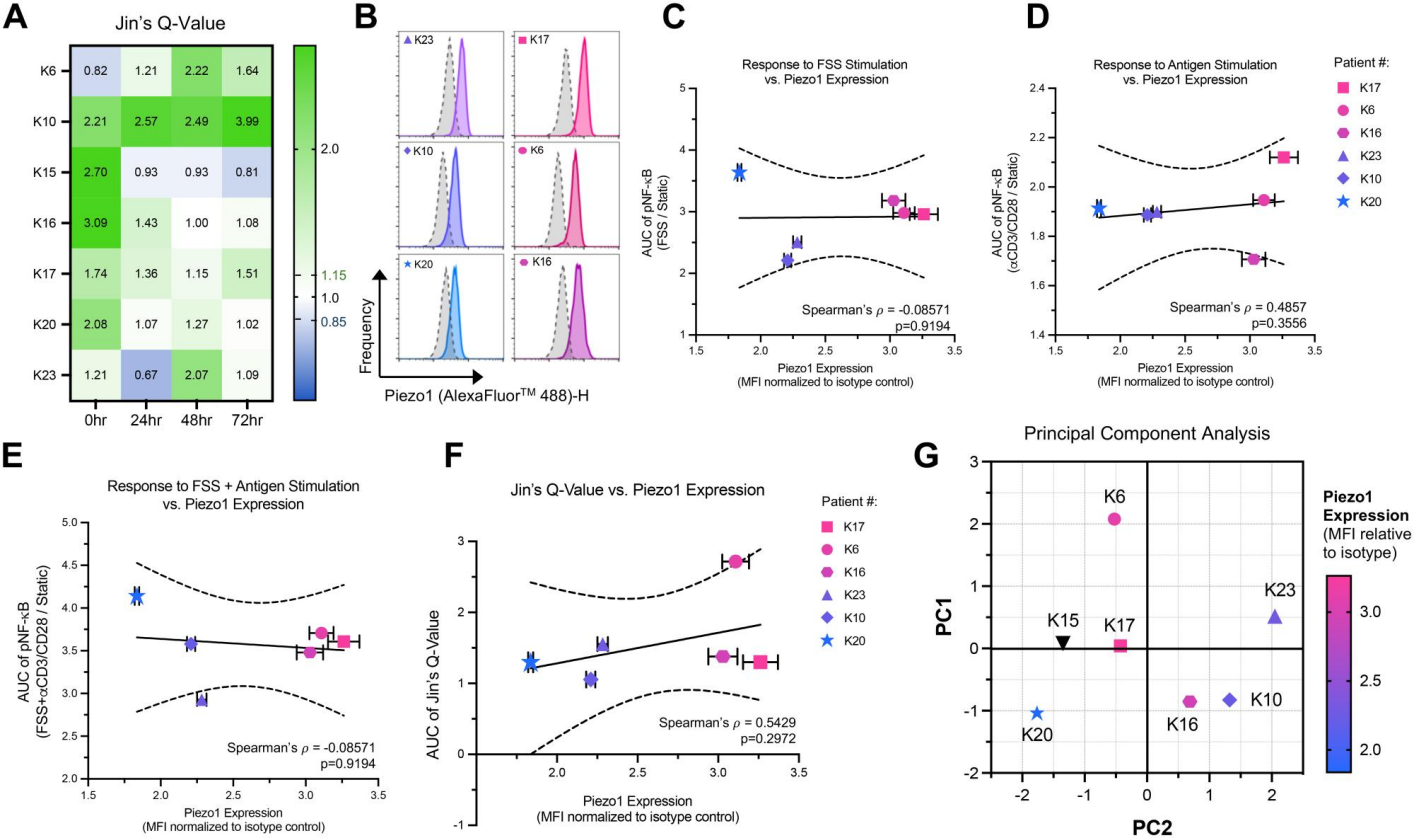
Synergy quantification and principal component analysis reveal donor-dependent mechanical and antigen-driven activation profiles. (A) Jin’s *Q* values were calculated from NF-κB phosphorylation data at 0, 24, 48, and 72 h to quantify the degree of synergy between FSS and αCD3/CD28 stimulation in patient-derived T cells. Jin’s *Q* value corresponds with an antagonistic (*Q* < 0.85), additive (0.85 ≤ *Q* ≥ 1.15), or synergistic (*Q* > 1.15) relationship. (B) Representative flow cytometry histograms of Piezo1 expression in T cells obtained from each patient compared with its corresponding isotype controls (dashed-gray data). (C–F) Correlation between Piezo1 expression (MFI normalized to isotype control) with the AUC of NF-κB phosphorylation for (C) FSS stimulation, (D) αCD3/CD28, (E) combined FSS and αCD3/CD28 stimulation, and (F) Jin’s *Q* value. (G) PCA of standardized AUC values for FSS, αCD3/CD28, combined stimulation, and Jin’s *Q* values. Each donor’s PCA score symbol was color coded according to relative Piezo1 expression levels (MFI normalized to isotype), where black (K15) indicates no Piezo1 expression measurement was obtained. (C–F) Spearman’s correlation coefficient (*ρ*), and *P* value were calculated to determine degree of correlation. The line of best fit was plotted with the 95% confidence interval for visualization purposes only. Horizontal error bars represent the standard error of the mean (SEM) of the Piezo1 expression values measured in triplicate.

To quantify the extent of synergy between FSS and antigen stimulation via αCD3/CD28 over time, Jin’s Q-value was calculated using NF-κB phosphorylation data collected across each timepoint (0, 24, 48, 72 h) for each patient (Fig. 4A). Most patient-derived T cells exhibited a Q-value greater than 1.15 and multiple timepoints, suggesting a synergistic relationship between FSS stimulation and αCD3/CD28 stimulation. However, the magnitude of synergy and the dynamic profile of how synergy changed over time varied greatly between donors. For example, the highest synergy was observed in T cells from patient K10 (*Q* = 4.0) at 72 h post-stimulation, whereas K15 (*Q* = 2.7) and K16 (*Q* = 3.1) peaked at 0 h post-stimulation. Importantly, since Jin’s synergy calculation assumes additive effects in each independent stimulation condition, the resulting *Q* value may underestimate activation in patient-derived T cells that respond strongly to FSS but weakly to antigen stimulation. To account for this, the fold-change in NF-κB phosphorylation (MFI relative to static control) is also shown for each patient to demonstrate the magnitude of activation induced by FSS, antigen, and combined stimulation (Fig. S1). For example, K16 exhibited a strong FSS-induced activation response despite minimal antigen-induced activation, resulting in a low *Q* value but high intrinsic mechanosensitivity.

Since previous work has demonstrated the important role of mechanosensitive calcium ion channels, such as Piezo1, in FSS-induced T cell activation, patient T cells were evaluated for Piezo1 expression prior to activation experiments. Basal Piezo1 expression was quantified in flow cytometry as the MFI normalized to the corresponding isotype control (Fig. 4B). To determine whether activation strength was associated with Piezo1 expression, Spearman’s correlation (*ρ*) analysis was performed between Piezo1 expression and the AUC of NF-κB phosphorylation across all timepoints. No significant correlations were observed for FSS alone (Fig. 4C; *ρ* =−0.0857, *P* = 0.919), αCD3/CD28 alone (Fig. 4D; *ρ*  = 0.486, *P* = 0.356), and combined FSS and αCD3/CD28 (Fig. 4E; *ρ* =−0.0857, *P* = 0.919) stimulation. Similarly, Piezo1 expression was correlated with the AUC of Jin’s *Q* value (Fig. 4F; *ρ*  = 0.543, *P* = 0.297) revealing no significant association. Together, these data suggest that basal Piezo1 expression is not solely responsible for the observed inter-donor differences in mechanical- and antigen-induced activation under these conditions.

PCA incorporating the standardized AUC values from FSS, αCD3/CD28, combined FSS and αCD3/CD28, and Jin’s Q value was performed as an exploratory approach to visualize patterns in activation responses across patients (Fig. 4G). The first principal component (PC1) accounted for 42.1% of the total variance and was primarily influenced by FSS and *Q* value, representing an axis for the mechanical and synergistic activation potential. Moreover, the second principal component (PC2) accounted for 25.3% of the total variation and was mainly influenced by CD3/CD28 stimulation, representing an axis for antigen-driven activation. While interpretation is limited by the small sample size, donor K6 was positioned very high on PC1, consistent with relatively higher FSS-associated responsiveness, whereas donors K23 and K10 were positioned high on PC2, suggesting a more antigen-driven activation profile. Interestingly, K6 exhibited relatively high Piezo1 expression, which aligns with its position on the PC1 axis indicating enhanced FSS responsiveness. Moreover, the highest points on the PC2 axis, K23 and K10, exhibited moderate to low Piezo1 expression, suggesting reduced association with mechanotransduction in these samples. In contrast, the most negative extreme for both PC1 and PC2, K20, exhibited the lowest relative Piezo1 expression, consistent with comparatively lower activation across all conditions.

## Discussion

**Figure 5:**
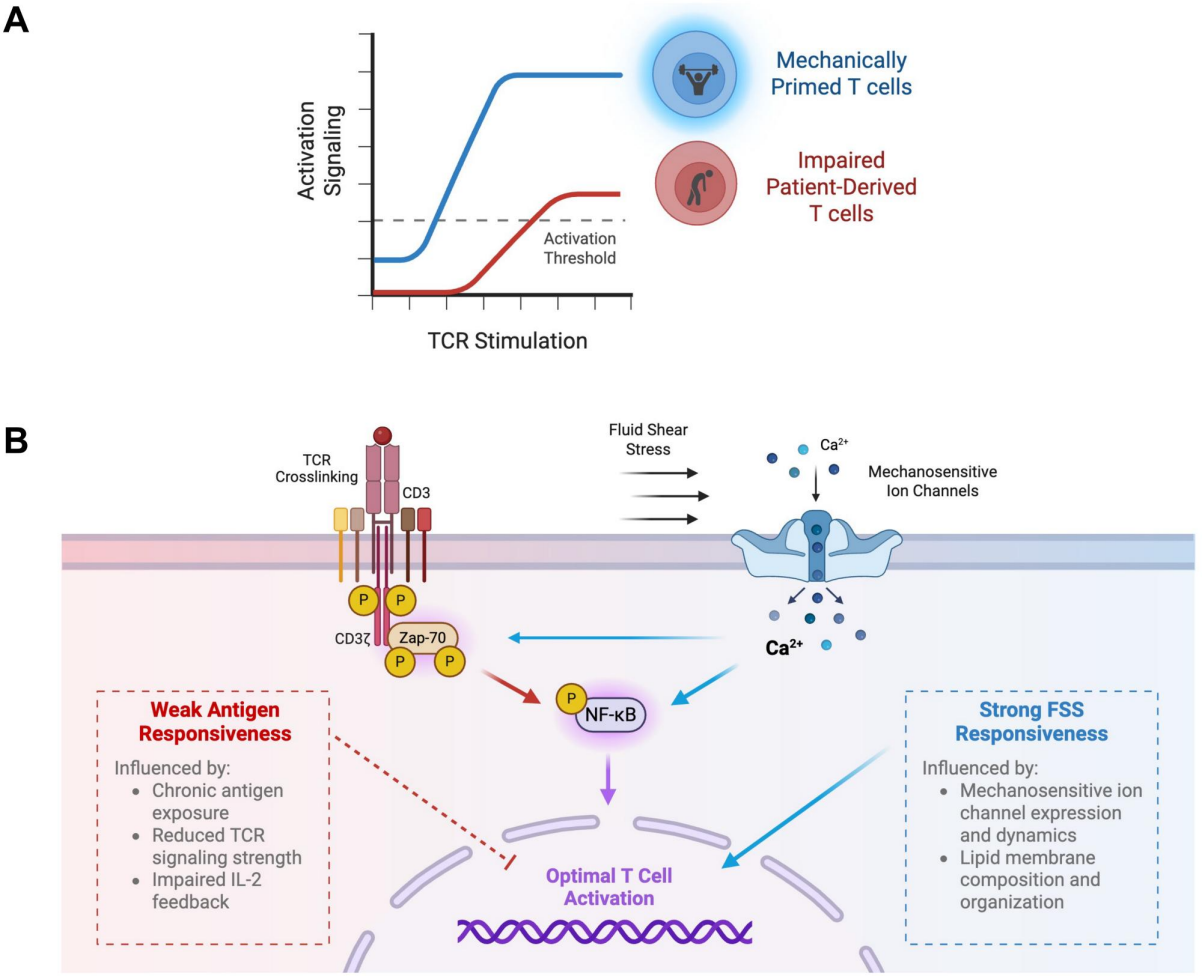
Fluid shear stress serves as a mechanical co-stimulatory cue to restore activation potential of patient-derived T cells. Graphical representation of mechanical priming via FSS to increase activation signaling beyond the activation threshold, compensating for weak antigen responsiveness often seen in patient-derived T cells. Made in BioRender.

To our knowledge, this study is the first to demonstrate that FSS can directly modulate activation signaling and effector function in T cells derived from patients with mCRPC. T cells were isolated from whole blood samples and were exposed to a constant and uniform FSS in cone-and-plate viscometers for 1 h in the presence and absence of biochemical activation via bead-bound αCD3/CD28. It was observed that FSS exposure consistently potentiated biochemical activation pathways, beginning with early activation signaling via enhanced NF-κB phosphorylation to increased pro-inflammatory cytokine production and activation marker expression. Furthermore, donor-dependent increases in CD4+ memory acquisition and CD25^+^/CD$25_{\text{hi}}^{+}$ upregulation were observed, reflecting enhanced T cell priming and activation. Collectively, these results demonstrate that FSS acts as a co-stimulatory cue that strengthens and sustains T cell activation through coordinated upregulation of signaling, functional, and phenotypic responses (Fig. 5). Importantly, comparison to age-matched healthy male donor T cells indicates that this co-stimulatory role of FSS is conserved but is more pronounced in mCRPC T cells where baseline TCR responsiveness is reduced.

To assess the mechanosensitivity of patient-derived T cells, NF-κB phosphorylation was quantified following FSS exposure. FSS independently activated NF-κB and synergistically enhanced signaling when combined with biochemical stimulation via αCD3/CD28. Importantly, αCD3/CD28 stimulation without FSS elicited only minimal NF-κB phosphorylation across all timepoints, indicating poor TCR-mediated activation in mCRPC patient-derived T cells. This weak activation response contrasts with previous observations in healthy donor T cells,^13^ where αCD3/CD28 stimulation induced robust NF-κB phosphorylation under identical experimental conditions. Consistent with these prior reports, the age-approximated healthy male cohort in this study also demonstrated strong and sustained NF-κB phosphorylation in response to αCD3/CD28 alone, with FSS further amplifying this response rather than rescuing it. However, the diminished activation capacity observed in cancer patient-derived T cells is consistent with well-documented impairments in TCR signaling associated with chronic antigen exposure, prior therapeutic interventions, and systemic immunosuppression associated with advanced disease.^17^^,^^22^^,^^24^ Together, these findings suggest that FSS serves a distinct functional role depending on baseline T cell fitness, where it acts primarily as an amplifier in healthy T cells, but as both an activator and amplifier in the mCRPC patient-derived T cells.

The TCR is recognized as a mechanosensor that exhibits enhanced activation signaling with increased application of force.^25^ These forces are generated and transmitted through the actin cytoskeleton, which organizes TCR clustering and sustains downstream signaling at the immunological synapse. Studies have shown that disruption of actin remodeling impairs TCR signal amplification, underscoring the requirement for coordinated cytoskeletal dynamics during activation.^26–28^ In addition, previous work, including our own, has demonstrated that FSS induces cytoskeletal remodeling across multiple cell types,^29–32^ including T cells.^13^ In this context, we evaluated F-actin following shear exposure to determine whether FSS-induced NF-κB activation occurred alongside changes in F-actin. In patient-derived T cells, FSS exposure did not reduce total F-actin content, whereas the combined FSS and αCD3/CD28 stimulation significantly increased F-actin signal, consistent with activation-associated actin remodeling rather than cytoskeletal collapse. Notably, the absence of a significant increase in F-actin signal under αCD3/CD28 stimulation alone further underscores the impaired activation capacity of patient-derived T cells. In contrast, healthy donor T cells exhibited robust activation signaling with αCD3/CD28 stimulation alone, suggesting that cytoskeletal remodeling may already be efficiently engaged in these cells without requiring additional mechanical input. Together, these data suggest that conventional biochemical activation with αCD3/CD28 alone may be insufficient to overcome reduced responsiveness in mCRPC patient-derived T cells, highlighting the potential value of using mechanical stimulation to enhance activation signaling.

To determine whether these FSS-induced signaling events translated into functional activation, we examined NF-κB dependent cytokine responses, focusing on the canonical targets IFN-γ, IL-2, and TNF-α as indicators of autocrine proliferative and effector inflammatory function. Notably, FSS stimulation alone was sufficient to increase intracellular cytokine synthesis but did not enhance surface receptor expression (CD69 and CD25). These findings are consistent with our previous work examining healthy donor T cells, which similarly showed that FSS alone can enhance intracellular cytokine production but is insufficient to induce cytokine secretion without TCR engagement.^13^ This selective response underscores the canonical TCR signaling checkpoints that are required to achieve full T cell activation, where transient activation of NF-κB and NFAT is sufficient to initiate cytokine gene transcription.^33–36^ However, the absence of TCR engagement likely prevents sustained transcription and autocrine IL-2 feedback necessary for CD25 and CD69 upregulation.^37^^,^^38^ Although additional transcription factor families, including interferon regulatory factors (IRFs), contribute to interferon-associated and differentiation programs in T cells, the present study was designed to evaluate the classical NF-κB dependent activation axis downstream of TCR-crosslinking. Furthermore, the absence of immunological synapse formation and polarized vesicle trafficking under mechanical stimulation likely accounts for the lack of efficient cytokine secretion.^39^ Together, these findings suggest that FSS alone may partially induce an activation state that primes T cells by lowering their activation threshold, enabling synergistic responses when combined with TCR-mediated stimulation.

Differences in baseline T cell phenotype, particularly in memory composition and CD25 expression, may further contribute to the observed variability in mechanosensitive responses. For instance, patient K6 retained a larger CD4^+^/CD45Ro^-^/CD25^-^ population following αCD3/CD28 stimulation and demonstrated a shear-associated increase in both CD45Ro memory conversion and CD25^+^/CD$25_{\text{hi}}^{+}$ upregulation. In contrast, K10 and K23 T cells were already predominantly CD45Ro^+^ and highly CD25-expressing under static αCD3/CD28 stimulation, limiting further increases in these percentage-based markers and suggesting ceiling effects at the 72 h timepoint. Notably, cytokine production in K10 was highest in the αCD3/CD28 with FSS condition despite minimal additional changes in proximal activation markers. This finding suggests that mechanical stimulation may enhance downstream functional output even when surface marker frequencies appear saturated, a pattern that has also been observed in prior studies with healthy donor T cells.^13^

Baseline heterogeneity may reflect differences in treatment exposure and clinical context at the time of sampling. K6 was sampled during ongoing Pluvicto therapy (Pre-C5), K23 prior to initiation of Pluvicto (Pre-C1), and K10 while receiving abiraterone with prednisone, each representing distinct treatment states that may influence differentiation and activation. Importantly, PSA levels differed across donors at the time of sampling, and peripheral CD4^+^ phenotype did not clearly align with clinical response in this small cohort. Notably, K6 exhibited a lower baseline CD45Ro^+^/CD25^+^ profile that more closely resembled the healthy donor T cells, reflecting a less differentiated and more phenotypically plastic subset. Despite this, K6 was clinically responsive to Pluvicto therapy, achieving PSA50 (defined as a ≥50% reduction in PSA from initiation of treatment) and favorable radiographic response. In contrast, K10 and K23 displayed predominantly CD45Ro^+^/CD25^+^ phenotypes at baseline, consistent with a more differentiated or chronically stimulated CD4^+^ T cell compartment. However, this phenotype may also reflect multiple states, such as activated effector/memory cells, regulatory T cells (Tregs), or more terminally differentiated populations, each of which could limit the magnitude of additional phenotypic shifts observed at this timepoint. These observations should therefore be interpreted cautiously, as baseline differences may relate to treatment timing, steroid exposure, disease burden, or other unmeasured factors. Taken together, these data suggest that FSS may differentially modulate TCR-driven responses depending on baseline differentiation state and activation status, with greater observable phenotypic shifts in less differentiated populations.

Although Piezo1 expression was detected in all patient-derived T cells, its abundance did not correlate with activation magnitude nor synergy over time, suggesting that mechanosensitivity in patient-derived T cells is not solely determined by Piezo1 expression levels. However, PCA revealed an interesting trend in the most extreme values on the principal component axes, where high PC1, high PC2, and low PC1/PC2 scores were associated with relatively high, moderate to low, and very low Piezo1 expression, respectively. Furthermore, the PCA accounted for approximately 67% of the total variance across donors, indicating that while force- and antigen-driven activation responses may explain the majority of the observed differences, there still remain additional unmeasured factors that contribute to the observed variability. These findings suggest that Piezo1 expression may contribute to, but is not solely responsible for, FSS-induced T cell activation.

Another possible mechanism for the variation in T cell mechanosensitivity may arise from differences in mechanosensitive ion channel gating dynamics driven by inherent lipid membrane composition, organization, and cytoskeletal architecture.^40–43^ It has been well established that mechanosensitive ion channel dynamics are influenced by membrane tension and curvature, where their probability of opening can be modulated by cholesterol content, association with lipid rafts, and local membrane stiffness.^44–48^ Furthermore, aberrant biophysical properties of immune cells have been reported in cancer and autoimmune diseases, which may further influence how mechanical forces are sensed and transmitted through mechanosensitive channels.

While this study provides new insights into the mechanotransduction of cancer patient-derived T cells, there are limitations that should be acknowledged. For instance, the sample size was limited to a small cohort of patients with mCRPC. Additional patient samples would be needed to fully capture the observed inter-patient variability and to evaluate correlations with clinical parameters, such as treatment history or disease progression. Furthermore, the 1 h FSS exposure during the initial αCD3/CD28 stimulation warrants a more comprehensive optimization to determine the impact of longer FSS exposure or repeated mechanical conditioning on T cell activation and persistence. The FSS magnitude of 5 dyn/cm^2^ was selected based on prior dose-response studies demonstrating a graded time-dependent (15–60 min) and magnitude-dependent (0.5–5.0 dyn/cm^2^) increase in ZAP70 phosphorylation^12^ and was consistent with our prior mechanotransduction studies in healthy donor T cells.^13^ Importantly, this magnitude also falls within the physiological range of wall shear stresses in the circulatory system, corresponding to the higher end of post-capillary venules and the lower end of arterioles, further supporting the biological relevance of this stimulus.^49^

This study population was heavily treated (median prior treatments = 4) and older (mean age = 73.6 yr), factors known to reduce immune cell responsiveness. Despite this limitation, we still saw evidence of the ability to activate T cells. Importantly, comparison to age-approximated healthy male donors shows that although baseline TCR-driven activation is reduced in mCRPC T cells, they remain mechanosensitive, supporting the translational potential of FSS in functionally impaired immune populations. Future work should replicate these studies earlier in the disease state and also investigate additional activation, differentiation, and exhaustion signaling pathways using techniques like single cell transcriptomics to gain a more comprehensive understanding of how mechanical forces reprogram T cell states and modulate their functional capacity.

Furthermore, while in vitro culture does not fully recapitulate the hemodynamic and biochemical complexity of the in vivo environment, controlled conditions were necessary to isolate the independent contribution of FSS to T cell activation. No exogenous cytokines were included so that mechanotransduction effects could be evaluated without biasing differentiation toward specific T cell subsets. Culture conditions were kept identical across all experimental groups to ensure that observed differences could be attributed to mechanical stimulation rather than culture environment.

Collectively, our findings demonstrate that FSS may act as a mechanical co-stimulus that synergizes with canonical TCR-mediated activation. Importantly, the translational goal of this platform is not necessarily to model in vivo physiology, but to inform ex vivo T cell activation and expansion strategies that are relevant to adoptive cell therapies. In the context of mCRPC and other solid tumors, where T cells are frequently exhibit dysfunction, exhaustion, or limited persistence, there remains a critical need for manufacturing approaches that can enhance T cell activation while preserving favorable phenotypes. Our results suggest that FSS may serve as a tunable input that could be integrated into ex vivo expansion protocols to restore activation capacity and potentially enhance therapeutic potency for adoptive cell therapies such as CAR-T, intra-tumoral immunotherapy, or TIL-based approaches. Additionally, there are currently a large number of immunotherapy approaches being studied in mCRPC, including, but not limited to, T cell redirecting bispecific antibodies targeting STEAP-1xCD3,^50^ KLK2xCD3^51^ constructs, B7-H3 targeted therapies,^52^ in situ activation approaches such as Syn-T,^53^^,^^54^ and multiple studies exploring ex vivo and in vivo T cell manufacturing.^55^^,^^56^ Within the evolving therapeutic landscape, this work could be a promising springboard for future research that aims to improve therapy outcomes in patients with advanced prostate cancer; FSS in vivo is an intriguing parameter to explore in future work as a variable that could explain differing outcomes across patients. Moreover, a deeper understanding of how patient-specific variables, such as age, treatment history, T cell differentiation state, and membrane composition influence mechanosensitivity may enable more personalized optimization of T cell-based therapies, ultimately strengthening therapeutic responses in patients with prostate cancer and other solid tumors.

## Supplementary Material

vkag166_Supplementary_Data

## Data Availability

Data, materials, and additional information are available upon request to the corresponding authors, mike.king@rice.edu and kerry.schaffer@vumc.org.

## Abbreviations

αCD3/CD28: Dynabeads^®^ Human T-Activator CD3/CD28 magnetic beads
ACT: Adoptive cell therapy
ADT: Androgen deprivation therapy
AMPAR: α-amino-3-hydroxy-5-methyl-4-isoxazolepropionic acid receptor
AUC: Area under the curve
BSA: Bovine serum albumin
CAR-T: Chimeric antigen receptor T cell
CMV: Cytomegalovirus
CTCF: Corrected total cell fluorescence
DC: Dendritic cell
FBS: Fetal bovine serum
FSS: Fluid shear stress
HbA1c: Hemoglobin A1c (glycated hemoglobin)
HBSS: Hank’s balanced salt solution
HBV: Hepatitis B virus
HCV: Hepatitis C virus
HIV: Human immunodeficiency virus
HTLV: Human T-lymphotropic virus
IRB: Institutional Review Board
IRF: Interferon regulatory factors
MFI: Median fluorescence intensity
mCRPC: Metastatic castration-resistant prostate cancer
MSI: Microsatellite instability
PBMC: Peripheral blood mononuclear cell
PCA: Principal component analysis
PC1/PC2: Principal component 1/Principal component 2
PFA: Paraformaldehyde
PS: Penicillin-streptomycin
PSA: Prostate specific antigen
Q-value (Jin’s Q): Synergy index calculated using Jin’s formula
RM-ANOVA: Repeated measures analysis of variance
ROI: Region of interest
RT: Room temperature
TCR: T cell receptor
TME: Tumor microenvironment
Tregs: Regulatory T cells
VICC: Vanderbilt-Ingram Cancer Center
yrs: Years

## References

[1] Rawla P. Epidemiology of prostate cancer. World J Oncol. 2019;10:63–89. 10.14740/wjon1191 31068988 PMC6497009

[2] Zhang AC , Rasul R, Golden A, Feuerstein MA. Incidence and mortality trends of metastatic prostate cancer: surveillance, epidemiology, and end results database analysis. Can Urol Assoc J. 2021;15:E637–E643. 10.5489/cuaj.7173 34171209 PMC8631846

[3] Zhang S , Zhang T, Kinsella GK, Curtin JF. A review of the efficacy of prostate cancer therapies against castration-resistant prostate cancer. Drug Discovery Today. 2025;30:104384. 10.1016/j.drudis.2025.104384 40409404

[4] López-Campos F et al Immunotherapy in advanced prostate cancer: current knowledge and future directions. Biomedicines. 2022;10:537. 10.3390/biomedicines10030537 35327339 PMC8945350

[5] Novysedlak R et al The immune microenvironment in prostate cancer: a comprehensive review. Oncology. 2025;103:521–545. 10.1159/000541881 39380471 PMC12140600

[6] Velho PI et al Nivolumab in patients with metastatic castration-resistant prostate cancer with and without DNA repair defects. Clin Cancer Res. 2024;30:5342–5352. 10.1158/1078-0432.ccr-24-1595 39330991

[7] Antonarakis ES et al Pembrolizumab for treatment-refractory metastatic castration-resistant prostate cancer: multicohort, open-label phase II KEYNOTE-199 study. J Clin Oncol. 2020;38:395–405. 10.1200/jco.19.01638 31774688 PMC7186583

[8] Petrylak DP et al Pembrolizumab plus docetaxel versus docetaxel for previously treated metastatic castration-resistant prostate cancer: the randomized, double-blind, phase III KEYNOTE-921 trial. J Clin Oncol. 2025;43:1638–1649. 10.1200/jco-24-01283 40043230 PMC12058370

[9] Sarna NS et al Immunomechanobiology: engineering the activation and function of immune cells with the mechanical signal of fluid shear stress. IEEE Rev Biomed Eng. 2025;18:231–249. 10.1109/rbme.2024.3505073 40030260

[10] Ghazanfari D , Ren L, Cantú MS, King MR. Cellular mechanoactivation of antigen-presenting cells and T cells for cancer immunotherapy. Curr Opin Biomed Eng. 2025;36:100619. 10.1016/j.cobme.2025.100619

[11] Pathni A , Wagh K, Rey-Suarez I, Upadhyaya A. Mechanical regulation of lymphocyte activation and function. J Cell Sci. 2024;137:jcs219030. 10.1242/jcs.219030 38995113 PMC11267459

[12] Hope JM et al Fluid shear stress enhances T cell activation through Piezo1. BMC Biol. 2022;20:61. 10.1186/s12915-022-01266-7 35260156 PMC8904069

[13] Sarna NS et al Enhanced and sustained T cell activation in response to fluid shear stress. iScience. 2024;27:109999. 10.1016/j.isci.2024.109999 38883838 PMC11177201

[14] Dombroski JA et al Fluid shear stress enhances dendritic cell activation. Immunobiology. 2023;228:152744. 10.1016/j.imbio.2023.152744 37729773 PMC10841200

[15] Mitchell MJ , Lin KS, King MR. Fluid shear stress increases neutrophil activation via platelet-activating factor. Biophys J. 2014;106:2243–2253. 10.1016/j.bpj.2014.04.001 24853753 PMC4052238

[16] Dombroski JA et al Activation of dendritic cells isolated from the blood of patients with prostate cancer by ex vivo fluid shear stress stimulation. Curr Protocols. 2023;3:e933. 10.1002/cpz1.933 PMC11178276 38047658

[17] Janelle V , Delisle J-S. T-cell dysfunction as a limitation of adoptive immunotherapy: current concepts and mitigation strategies. Cancers. 2021;13:598. 10.3390/cancers13040598 33546277 PMC7913380

[18] Chow A , Perica K, Klebanoff CA, Wolchok JD. Clinical implications of T cell exhaustion for cancer immunotherapy. Nat Rev Clin Oncol. 2022;1–16. 10.1038/s41571-022-00689-z 36216928 PMC10984554

[19] Jin Z. About the evaluation of drug combination. Acta Pharmacologica Sinica. 2004;25:146–147. 14769200

[20] Pedregosa F et al Scikit-learn: machine learning in python. J Machine Learn Res. 2011;12:2825–2830.

[21] Kagamu H et al CD4+ T-cell immunity in the peripheral blood correlates with response to anti-PD-1 therapy. Cancer Immunol Res. 2020;8:334–344. 10.1158/2326-6066.cir-19-0574 31871122

[22] Venkatesh H , Fong L. CD4+ T cell dysfunction in cancer. J Exp Med. 2025;222:e20241417. 10.1084/jem.20241417 40772965

[23] Inomata M et al Peripheral CD4 memory T cells predict the efficacy of immune checkpoint inhibitor therapy in patients with non-small cell lung cancer. Sci Rep. 2023;13:10807. 10.1038/s41598-023-37736-3 37402763 PMC10319808

[24] McLane LM , Abdel-Hakeem MS, Wherry EJ. CD8 T cell exhaustion during chronic viral infection and cancer. Annu Rev Immunol. 2015;37:1–39. 10.1146/annurev-immunol-041015-055318 30676822

[25] Jeffreys N et al Mechanical forces amplify TCR mechanotransduction in T cell activation and function. Appl Phys Rev. 2024;11:011304. 10.1063/5.0166848 38434676 PMC10848667

[26] Kumari S et al Cytoskeletal tension actively sustains the migratory T‐cell synaptic contact. EMBO J. 2020;39:EMBJ2019102783. 10.15252/embj.2019102783 PMC7049817 31894880

[27] Hui KL , Balagopalan L, Samelson LE, Upadhyaya A. Cytoskeletal forces during signaling activation in Jurkat T-cells. Mol Biol Cell. 2015;26:685–695. 10.1091/mbc.e14-03-0830 25518938 PMC4325839

[28] Hui KL , Upadhyaya A. Dynamic microtubules regulate cellular contractility during T-cell activation. Proc Natl Acad Sci. 2017;114:E4175–E4183. 10.1073/pnas.1614291114 28490501 PMC5448208

[29] McCue S , Noria S, Langille BL. Shear-induced reorganization of endothelial cell cytoskeleton and adhesion complexes. Trends Cardiovasc Med. 2004;14:143–151. 10.1016/j.tcm.2004.02.003 15177265

[30] Kuo Y-C et al Oscillatory shear stress mediates directional reorganization of actin cytoskeleton and alters differentiation propensity of mesenchymal stem cells. Stem Cells. 2015;33:429–442. 10.1002/stem.1860 25302937

[31] Mu W et al Fluid shear stress upregulates E-Tmod41 via miR-23b-3p and contributes to F-actin cytoskeleton remodeling during erythropoiesis. PLoS One. 2015;10:e0136607. 10.1371/journal.pone.0136607 26308647 PMC4550387

[32] Son K , Hussain A, Sehmi R, Janssen L. The cycling of intracellular calcium released in response to fluid shear stress is critical for migration-associated actin reorganization in eosinophils. Cells. 2021;10:157. 10.3390/cells10010157 33467432 PMC7829934

[33] Shatrova AN et al Time-dependent regulation of IL-2R α-chain (CD25) expression by TCR signal strength and IL-2-induced STAT5 signaling in activated human blood T lymphocytes. PLoS One. 2016;11:e0167215. 10.1371/journal.pone.0167215 27936140 PMC5172478

[34] Marangoni F et al The transcription factor NFAT exhibits signal memory during serial T cell interactions with antigen-presenting cells. Immunity. 2013;38:237–249. 10.1016/j.immuni.2012.09.012 23313588 PMC3582823

[35] Dolmetsch RE , Lewis RS, Goodnow CC, Healy JI. Differential activation of transcription factors induced by Ca2+ response amplitude and duration. Nature. 1997;386:855–858. 10.1038/386855a0 9126747

[36] Fisher WG , Yang P-C, Medikonduri RK, Jafri MS. NFAT and NFκB activation in T lymphocytes: a model of differential activation of gene expression. Ann Biomed Eng. 2006;34:1712–1728. 10.1007/s10439-006-9179-4 17031595 PMC1764593

[37] Fuhrmann F et al Adequate immune response ensured by binary IL-2 and graded CD25 expression in a murine transfer model. eLife. 2016;5:e20616. 10.7554/elife.20616 28035902 PMC5201416

[38] Kalia V , Sarkar S. Regulation of effector and memory CD8 T cell differentiation by IL-2—a balancing act. Front Immunol. 2018;9:2987. 10.3389/fimmu.2018.02987 30619342 PMC6306427

[39] Griffiths GM , Tsun A, Stinchcombe JC. The immunological synapse: a focal point for endocytosis and exocytosis. J Cell Biol. 2010;189:399–406. 10.1083/jcb.201002027 20439993 PMC2867296

[40] Al-Aghbar MA , Jainarayanan AK, Dustin ML, Roffler SR. The interplay between membrane topology and mechanical forces in regulating T cell receptor activity. Commun Biol. 2022;5:40. 10.1038/s42003-021-02995-1 35017678 PMC8752658

[41] Robinson GA , Waddington KE, Pineda-Torra I, Jury EC. Transcriptional regulation of T-cell lipid metabolism: implications for plasma membrane lipid rafts and T-cell function. Front Immunol. 2017;8:1636. 10.3389/fimmu.2017.01636 29225604 PMC5705553

[42] Chubinskiy-Nadezhdin VI , Negulyaev YA, Morachevskaya EA. Cholesterol depletion-induced inhibition of stretch-activated channels is mediated via actin rearrangement. Biochem Biophys Res Commun. 2011;412:80–85. 10.1016/j.bbrc.2011.07.046 21798240

[43] Morachevskaya E , Sudarikova A, Negulyaev Y. Mechanosensitive channel activity and F‐actin organization in cholesterol‐depleted human leukaemia cells. Cell Biol Int. 2007;31:374–381. 10.1016/j.cellbi.2007.01.024 17317227

[44] Lewis AH , Grandl J. Mechanical sensitivity of Piezo1 ion channels can be tuned by cellular membrane tension. eLife. 2015;4:e12088. 10.7554/elife.12088 26646186 PMC4718726

[45] Beverley KM , Levitan I. Cholesterol regulation of mechanosensitive ion channels. Front Cell Dev Biol. 2024;12:1352259. 10.3389/fcell.2024.1352259 38333595 PMC10850386

[46] Lakk M et al Membrane cholesterol regulates TRPV4 function, cytoskeletal expression, and the cellular response to tension. J Lipid Res. 2021;62:100145. 10.1016/j.jlr.2021.100145 34710431 PMC8633027

[47] Yang S et al Membrane curvature governs the distribution of Piezo1 in live cells. Nat Commun. 2022;13:7467. 10.1038/s41467-022-35034-6 36463216 PMC9719557

[48] Yang X et al Structure deformation and curvature sensing of PIEZO1 in lipid membranes. Nature. 2022;604:377–383. 10.1038/s41586-022-04574-8 35388220

[49] Hossain MMN et al Angiogenic microvascular wall shear stress patterns revealed through three-dimensional red blood cell resolved modeling. Function. 2023;4:zqad046. 10.1093/function/zqad046 37753184 PMC10519277

[50] Kelly WK et al Xaluritamig, a STEAP1 × CD3 XmAb 2 + 1 immune therapy for metastatic castration-resistant prostate cancer: results from dose exploration in a first-in-human study. Cancer Discov. 2023;14:76–89. 10.1158/2159-8290.cd-23-0964 PMC10784743 37861461

[51] Stein MN et al Pasritamig, a first-in-class, bispecific T-cell engager targeting human kallikrein 2, in metastatic castration-resistant prostate cancer: a phase I study. J Clin Oncol. 2025;43:2515–2526. 10.1200/jco-25-00678 40450573 PMC12288886

[52] Sankyo DA. Clinical study of Ifinatamab Deruxtecan (I-DXd) in people with metastatic prostate cancer (MK-2400-001). NCT06925737. 2025 Apr 13.

[53] Syncromune, Inc. Phase 1 Trial of SYNC-T - Immunotherapy for advanced/metastatic castration-resistant prostate cancer. NCT05544227. 2022 Sept 16.

[54] Syncromune, Inc. A Study of SYNC-T Therapy SV-102 in participants with metastatic castration-resistant prostate cancer. NCT06533644. 2024 Aug 1.

[55] Dorff TB et al PSCA-CAR T cell therapy in metastatic castration-resistant prostate cancer: a phase 1 trial. Nat Med. 2024;30:1636–1644. 10.1038/s41591-024-02979-8 38867077 PMC11186768

[56] Pinto E et al From ex vivo to in vivo chimeric antigen T cells manufacturing: new horizons for CAR T-cell based therapy. J Transl Med. 2025;23:10. 10.1186/s12967-024-06052-3 39755643 PMC11700462

