# Supplementary material for: Fluid shear stress as a co-stimulatory cue to enhance T cell priming and restore activation in cancer patient T cells
Source: J Immunol. 2026 Aug 6;215(8):vkag166. doi: 10.1093/jimmun/vkag166 (PMC13445966; doi:10.1093/jimmun/vkag166)
Supplement: Supplementary file 1 [file vkag166_supplementary_data.pdf]

Supplemental Information:

Fluid shear stress as a co-stimulatory cue to enhance T cell priming and restore activation

Nicole S Sarna[*,#], Monika Antunovic[†,‡], Paula J Hurley[†,‡], Kerry R Schaffer[†,‡,§,¶], and Michael R King[*,#,¶]

[*] *Department of Bioengineering, Rice University, Houston, Texas, United States*

[#] *Department of Biomedical Engineering, Vanderbilt University, Nashville, Tennessee, United States*

[†] *Department of Medicine, Vanderbilt University Medical Center, Nashville, Tennessee, United States*

[‡] *Vanderbilt-Ingram Cancer Center, Nashville, Tennessee, United States*

[§] *Tennessee Valley Healthcare System, Nashville, Tennessee, United States*

[¶] *Corresponding author*

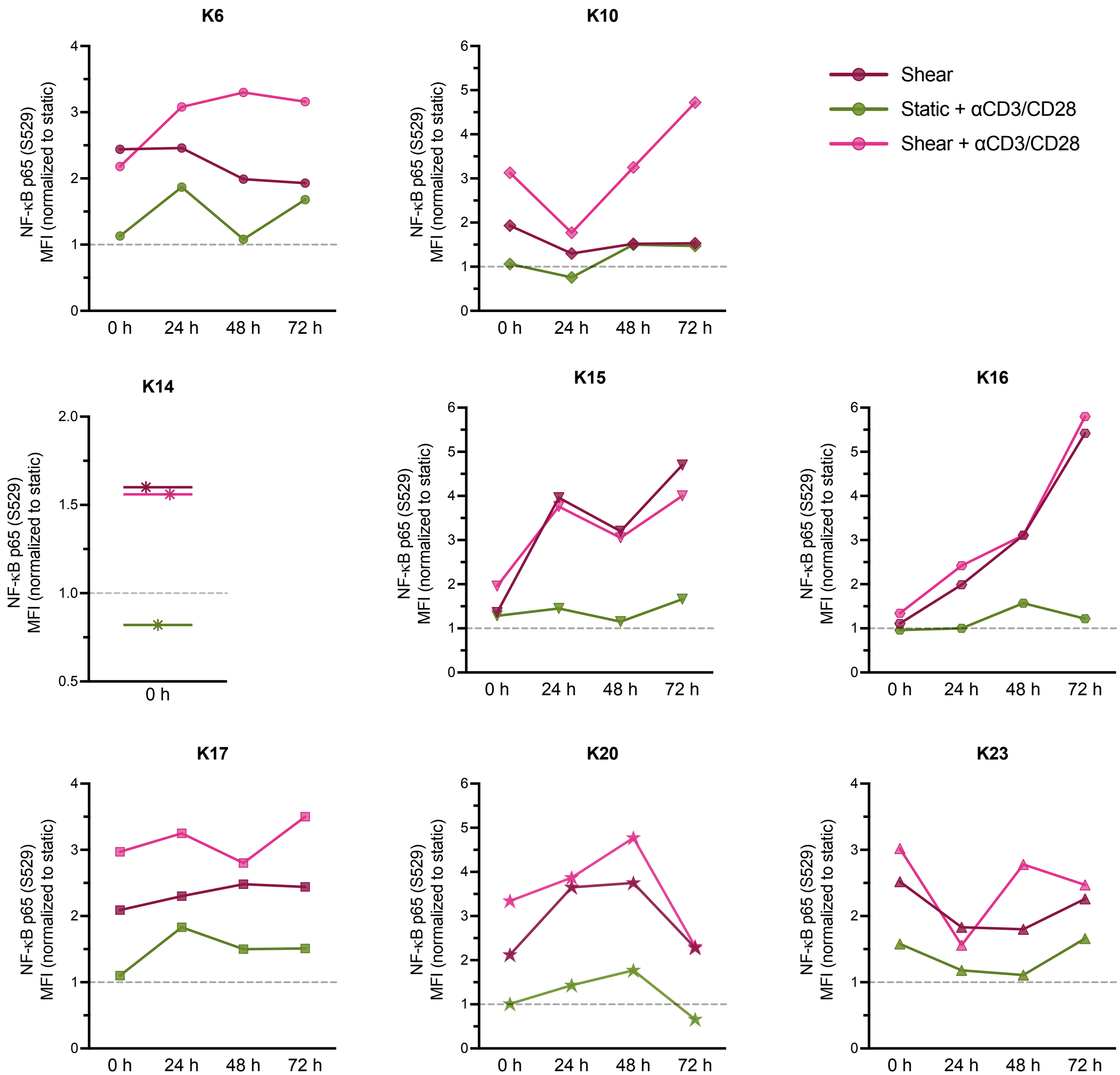

Supplementary Figure 1. Donor-dependent NF-κB phosphorylation in mCRPC patient-derived T cells following fluid shear stress and antigen stimulation. NF-κB phosphorylation (MFI) normalized to static control for each unique mCRPC patient and plotted as fold-change across timepoints (0, 24, 48, 72 h) for shear (FSS), αCD3/CD28, and combined stimulation conditions.

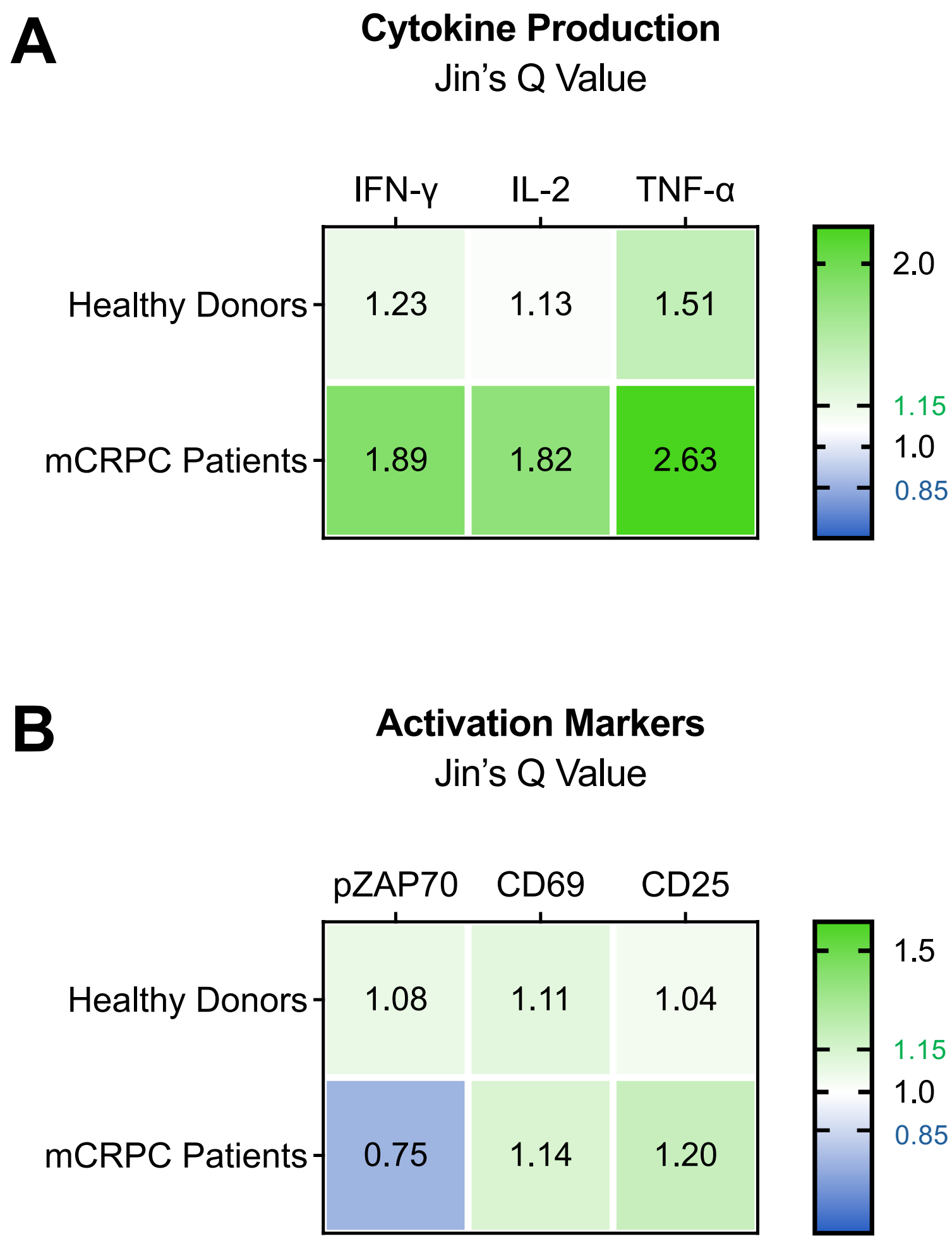

Supplementary Figure 2. Synergy between antigen and fluid shear stress stimulation in healthy donor and mCRPC patient-derived T cells.
(**A-B**) Heatmap representation of Jin's Q values for (**A**) cytokine production (IFN-γ, IL-2, TNF-α) and (**B**) activation markers (pZAP70, CD69, and CD25) following combined fluid shear stress and αCD3/CD28 stimulation relative to each condition alone in T cells isolated from elderly healthy male donors (n=3) and mCRPC patients (n=3). Jin's Q values were calculated to quantify the relationship between fluid shear stress and antigen stimulation, where $Q > 1.15$ indicates synergistic effects, $0.85 \leq Q \leq 1.15$ indicates additive effects, and $Q < 0.85$ indicates antagonism.

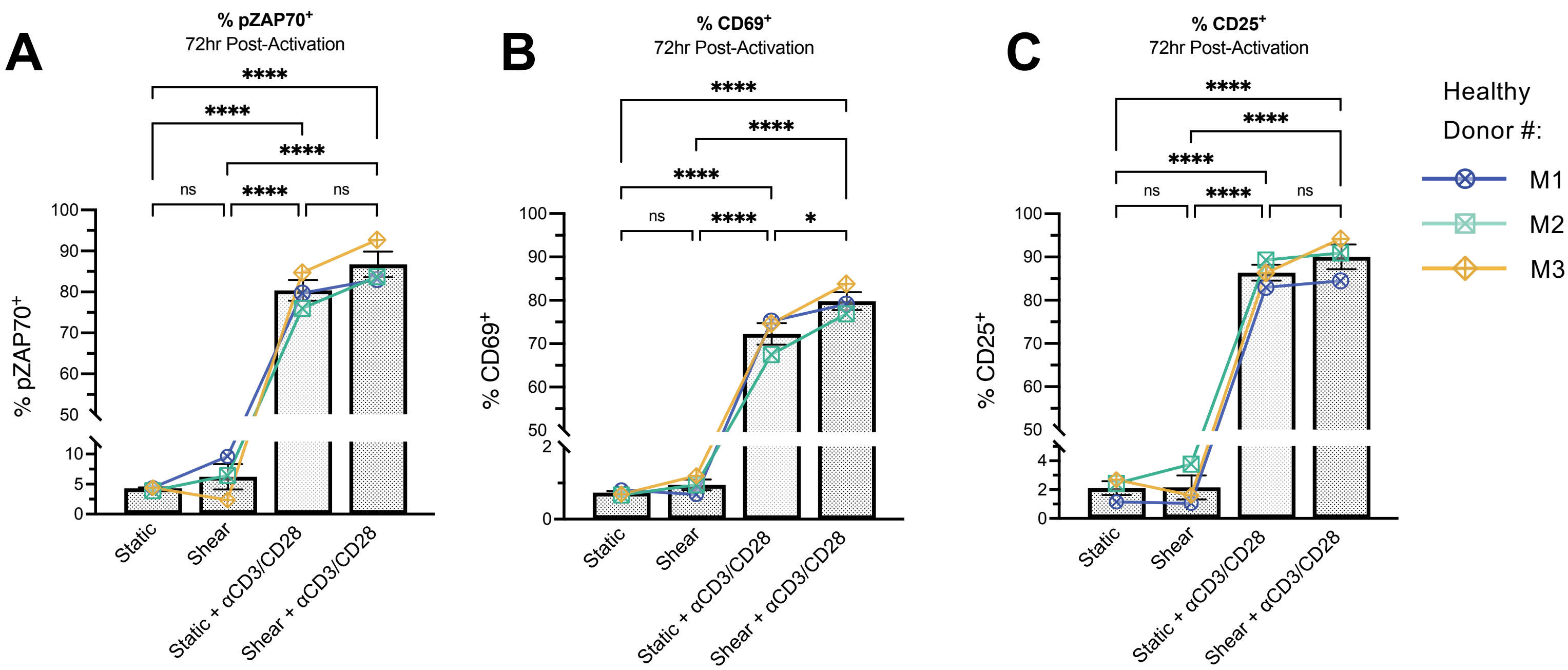

Supplementary Figure 3. Healthy donor T cell activation following antigen stimulation in the presence and absence of fluid shear stress. (**A-C**) Percentage of (**A**) pZAP70+, (**B**) CD69+, and (**C**) CD25+ in T cells isolated from elderly healthy male donors (n=3) 72 h post-activation. Individual donor responses are shown with connecting lines and bars represent mean ± SEM. Statistical comparisons were performed using repeated-measures one-way ANOVA with Tukey's multiple comparisons test. No significance (ns), *$p < 0.05$, ****$p < 0.0001$.

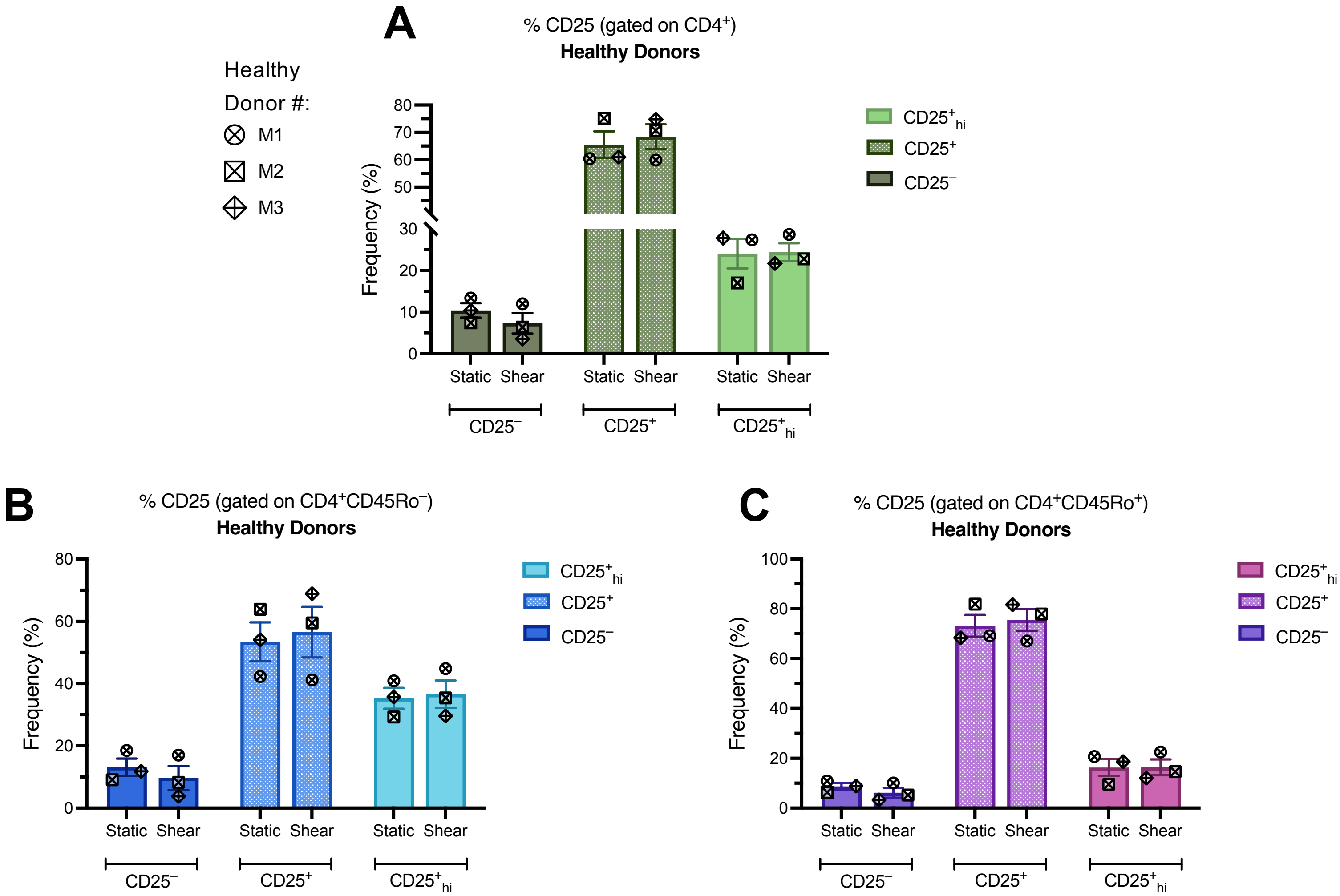

Supplementary Figure 4. Fluid shear stress minimally alters CD25 subset distribution in activated CD4⁺ T cells from elderly healthy male donors. (**A**) Distribution of CD25⁻, CD25⁺, and $CD25^{+}_{hi}$ populations within CD4⁺ T cells from unique healthy donors (n=3) 72 h following αCD3/CD28 stimulation under static and shear conditions. (**B**) CD25 subset distribution within CD4⁺CD45Ro⁺ (memory) T cells and (**C**) CD4⁺CD45Ro⁻ (naïve-like) T cells. Each point represents an individual donor, and bars indicate mean ± SEM. Paired ratio t-tests revealed no statistically significant differences between static and shear conditions.
